# Environmental fate and exposure; neonicotinoids and fipronil

**DOI:** 10.1007/s11356-014-3332-7

**Published:** 2014-08-07

**Authors:** J.-M. Bonmatin, C. Giorio, V. Girolami, D. Goulson, D. P. Kreutzweiser, C. Krupke, M. Liess, E. Long, M. Marzaro, E. A. D. Mitchell, D. A. Noome, N. Simon-Delso, A. Tapparo

**Affiliations:** 1 Centre National de la Recherche Scientifique, Centre de Biophysique Moléculaire, Rue Charles Sadron, 45071 Orléans cedex 02, France; 2 Department of Chemistry, University of Cambridge, Lensfield Road, CB2 1EW Cambridge, UK; 3 Dipartimento di Agronomia Animali Alimenti Risorse Naturali e Ambiente, Università degli Studi di Padova, Agripolis, viale dell’ Università 16, 35020 Legnaro, Padova Italy; 4 School of Life Sciences, University of Sussex, Falmer, Sussex, BN1 9QG UK; 5 Canadian Forest Service, Natural Resources Canada, 1219 Queen Street East, Sault Ste Marie, ON Canada P6A 2E5; 6 Department of Entomology, Purdue University, West Lafayette, IN 47907-2089 USA; 7 Department of System-Ecotoxicology, Helmholtz Centre for Environmental Research - UFZ, 04318 Leipzig, Germany; 8 Laboratory of Soil Biology, University of Neuchatel, Rue Emile Argand 11, 2000 Neuchatel, Switzerland; 9 Jardin Botanique de Neuchâtel, Chemin du Perthuis-du-Sault 58, 2000 Neuchâtel, Switzerland; 10 Task Force on Systemic Pesticides, Pertuis-du-Sault, 2000 Neuchâtel, Switzerland; 11 Kijani, Kasungu National Park, Private Bag 151, Lilongwe, Malawi; 12 Environmental Sciences, Copernicus Institute, Utrecht University, Heidelberglaan 2, 3584 CS Utrecht, The Netherlands; 13 Dipartimento di Scienze Chimiche, Università degli Studi di Padova, via Marzolo 1, 35131 Padova, Italy

**Keywords:** Neonicotinoid, Fipronil, Water, Soil, Dust, Plant, Guttation, Pollen, Nontarget, Bee, Invertebrates, Vertebrates

## Abstract

Systemic insecticides are applied to plants using a wide variety of methods, ranging from foliar sprays to seed treatments and soil drenches. Neonicotinoids and fipronil are among the most widely used pesticides in the world. Their popularity is largely due to their high toxicity to invertebrates, the ease and flexibility with which they can be applied, their long persistence, and their systemic nature, which ensures that they spread to all parts of the target crop. However, these properties also increase the probability of environmental contamination and exposure of nontarget organisms. Environmental contamination occurs via a number of routes including dust generated during drilling of dressed seeds, contamination and accumulation in arable soils and soil water, runoff into waterways, and uptake of pesticides by nontarget plants via their roots or dust deposition on leaves. Persistence in soils, waterways, and nontarget plants is variable but can be prolonged; for example, the half-lives of neonicotinoids in soils can exceed 1,000 days, so they can accumulate when used repeatedly. Similarly, they can persist in woody plants for periods exceeding 1 year. Breakdown results in toxic metabolites, though concentrations of these in the environment are rarely measured. Overall, there is strong evidence that soils, waterways, and plants in agricultural environments and neighboring areas are contaminated with variable levels of neonicotinoids or fipronil mixtures and their metabolites (soil, parts per billion (ppb)-parts per million (ppm) range; water, parts per trillion (ppt)-ppb range; and plants, ppb-ppm range). This provides multiple routes for chronic (and acute in some cases) exposure of nontarget animals. For example, pollinators are exposed through direct contact with dust during drilling; consumption of pollen, nectar, or guttation drops from seed-treated crops, water, and consumption of contaminated pollen and nectar from wild flowers and trees growing near-treated crops. Studies of food stores in honeybee colonies from across the globe demonstrate that colonies are routinely and chronically exposed to neonicotinoids, fipronil, and their metabolites (generally in the 1–100 ppb range), mixed with other pesticides some of which are known to act synergistically with neonicotinoids. Other nontarget organisms, particularly those inhabiting soils, aquatic habitats, or herbivorous insects feeding on noncrop plants in farmland, will also inevitably receive exposure, although data are generally lacking for these groups. We summarize the current state of knowledge regarding the environmental fate of these compounds by outlining what is known about the chemical properties of these compounds, and placing these properties in the context of modern agricultural practices.

## Introduction

Currently licensed for the management of insect pests in more than 120 countries, the class of insecticides known as neonicotinoids represent some of the most popular and widely used insecticides in the world (Jeschke et al. 2011; Van der Sluijs et al. 2013; Simon-Delso et al. 2014, this issue). Neonicotinoids are an acetylcholine-interfering neurotoxic class of insecticides (Matsuda et al. 2005) that are utilized in a variety of venues ranging from veterinary medicine, urban landscaping, and use in many agricultural systems as agents of crop protection. They can be applied by multiple methods as foliar sprays to above-ground plants, as root drenches to the soil, or as trunk injections to trees. However, it is estimated that approximately 60 % of all neonicotinoid applications globally are delivered as seed/soil treatments (Jeschke et al. 2011).

A key characteristic distinguishing neonicotinoids from other currently popular insecticide classes is their systemic nature. Neonicotinoids are relatively small molecules and are highly water soluble. Upon uptake by the plant, these compounds and their metabolites circulate (primarily via xylem transport) throughout plant tissues and provide a period of protection against a number of sap-feeding insects/arthropods (Nauen et al. 2008; Magalhaes et al. 2009). This systemic action is a key characteristic of the neonicotinoids and also fipronil, a phenylpyrazole insecticide largely used for crop protection that allows for great flexibility in methods of application. Additionally, neonicotinoids and fipronil are highly toxic to many classes of insects and exhibit relatively low vertebrate toxicity when compared with other insecticide classes currently in use (US EPA 2003). Therefore, these compounds are able to act specifically on insect pests while reducing impacts on some nontarget organisms (Tomizawa and Casida 2003, 2005; Tingle et al. 2003). However, in the last decade, concerns regarding the environmental fate and effects of these compounds—including soil persistence, effects on managed and wild pollinator species and other nontarget invertebrates, and the potential for contamination of untreated areas during sowing of treated seeds—have highlighted some of the pitfalls associated with the widespread use of these synthetic pesticides (Goulson 2013). Most recently, acute intoxication sources for bees associated with the use of seed-coating insecticides have been identified, specifically via contaminated guttation droplets (Girolami et al. 2009; Tapparo et al. 2011) and direct exposure of flying bees to dusts emitted by the drilling machine during sowing of treated seeds (Girolami et al. 2012; Krupke et al. 2012; Tapparo et al. 2012). Given the increasing evidence that these systemic insecticides pose serious risk of impacts on some nontarget organisms (Bijleveld van Lexmond et al. 2014, this issue), a review and synthesis of the literature describing the environmental fate and routes of exposure for these compounds is warranted.

## Chemical properties

### Volatility (air)

None of the systemic pesticides considered in this assessment (the neonicotinoids and fipronil) have a high vapor pressure. In general, values range between 2.8 × 10^−8^ and 0.002 mPa at 25 °C for these compounds. The low potential for volatilization of these substances indicates that these pesticides will most likely only be present in gaseous form for a short period during spray applications.

### Sorption to soil particles (soil)

Neonicotinoids and fipronil can bind to soil particles and this reduces their potential to be leached through the soil profile. Imidacloprid sorption was found to correlate positively to soil organic matter and mineral clay content, while desorption was lower at low temperature and at low pesticide concentration (Cox et al. 1997, 1998a, b, c; Broznic and Milin 2012; Broznic et al. 2012). The comparative study of four soils of contrasted texture and a reference sandy column revealed 27 to 69 % of imidacloprid leaching (97 % in the sand column) (Selim et al. 2010). Lowest mobility was observed in the soil with highest organic matter content (3.5 %), an effect attributed to the existence of hydrophilic bonding on functional groups of the pesticide which may bind to the phenolic hydroxyl and carboxylic acidic groups of soil organic matter. Studies on the effects of peat and tannic acid on mobility illustrate the importance of organic matter quality on imidacloprid dynamics in soil (Flores-Céspedes et al. 2002). Sorption coefficients differ between fibronil and its metabolites (desulfinyl, sulfide, and sulfone) (Ying and Kookana 2006). Neonicotinoids and fipronil and their metabolites also bind to particles in sediments that form the floor of freshwater and marine water bodies (e.g., Bobe et al. 1997; Baird et al. 2013). Bobe et al. (1997) observed that fipronil residues move from water to sediment within 1 week of application.

### Solubility (water)

In general terms, the systemic activity of compounds increases with increasing solubility due to improved uniformity in the distribution of the active ingredient in the formulation (Koltzenburg et al. 2010) and increased bioavailability of the pesticide (Pierobon et al. 2008). Transport and translocation are positively correlated with solubility (Chamberlain 1992). The solubility of neonicotinoids in water depends on multiple factors such as water temperature and pH as well as the physical state of the pesticide applied. The molecular weight of the neonicotinoids is between 250 and 300 g/mol, and solubility ranges between 184 (moderate) and 590.000 mg/L (high) for thiacloprid and nitenpyram, respectively, at 20 °C and at pH 7 (Carbo et al. 2008; Jeschke et al. 2011; PPDB 2012) (Table 1). When compared to the neonicotinoids, fipronil has a low solubility at 3.78 mg/L under the same conditions and has a larger molecular weight (437.15 g/mol) (Tingle et al. 2003). However, even lower solubilities ranging between 1.90 and 2.40 mg/L at pH 5 and pH 9, respectively were also reported.

**Table 1:** Leaching properties of various systemic insecticides (PPDB 2012)

Insecticide	Solubility in water at 20 °C at pH 7 (mg/L)	GUS leaching potential index	Aqueous photolysis DT50 (days) at pH 7	Water-sediment DT50 (days)
Acetamiprid	2,950 (high)	0.94 (very low)	34 (stable)	–
Clothianidin	340 (moderate)	4.91 (very high)	0.1 (fast)–Stable^a^	56.4 (moderately fast)
Dinotefuran	39,830 (high)	4.95 (very high)	0.2 (fast)	–
Fipronil	3.78 (low)	2.45 (moderate)	0.33 (fast)	68 (moderately fast)
Imidacloprid	610 (high)	3.76 (high)	0.2 (fast)	129 (slow)
Nitenpyram	590,000 (high)	2.01 (moderate)	–	–
Thiacloprid	184 (moderate)	1.44 (low)	Stable	28 (fast)
Thiamethoxam	4,100 (high)	3.82 (high)	2.7 (moderately fast)	40 (moderately fast)

^a^USEPA (2010)

It should be noted that commercial formulations often contain additional substances that alter the behavior of the active substance. For example, certain copolymers are used to increase the solubility or systemicity of fipronil (Dieckmann et al. 2010a, b, c) (US patents). In an experiment to determine leaching behavior, Gupta et al. (2002) consistently found commercially available formulas to have a higher leaching potential than analytical grade imidacloprid. This may be explained by the added surfactants, which keep the insecticide soluble or suspended for a longer period of time.

## Environmental fate—abiotic

### Air—environmental exposure by neonicotinoid and fipronil, contaminated dust

Seed coating/dressing is the leading delivery method for neonicotinoids in agriculture throughout the world. This method of pesticide application was initially considered to be a “safer” option for minimizing impacts on nontarget organisms by reducing drift (Ahmed et al. 2001; Koch et al. 2005). While it seems counterintuitive that environmental contamination could result from the use of treated seeds, mounting evidence indicates that the liberation of pesticides applied to seeds can and does arise via this widely used application method. We review research that has focused upon the dust generated during the sowing of neonicotinoid-treated seeds and highlight the risk of acute toxicity posed to honeybees that encounter dispersing dust. We further review current efforts to mitigate the drift of these compounds to nontarget areas.

#### History and background

Concerns regarding pesticide-contaminated dust from neonicotinoid- or fipronil-treated seeds originated from reports of atypical levels of honeybee losses in several countries following the planting of treated maize in spring. These incidents have been reported in Italy, France, Slovenia, Germany, USA, and Canada dating as far back as 1999 and as recently as 2013 (Greatti et al. 2003; Pistorius et al. 2009; Krupke et al. 2012; Van der Geest 2012; PMRA 2013). In all cases, a great number of dead and dying bees were found near the hive entrance. Many of these bees were foragers; however, in incidents reported in the USA in 2010 and 2011, many of the dead bees had the characteristic pubescence associated with newly eclosed nurse bees (C. Krupke, unpublished data) and neonicotinoids used in seed treatments were consistently found in pollen stored in affected hives (Krupke et al. 2012). Given that bee deaths have occurred in conjunction with the sowing of treated seeds, much attention has focused on possible routes of exposure for honeybees, both during and shortly after the planting period.

Contaminated dust was first implicated as a potential route of honeybee exposure to neonicotinoid residues following a study by Greatti et al. (2003). This work demonstrated that high levels of neonicotinoid-active ingredients occurred in the exhaust of modern pneumatic planters during seed sowing, and the same active ingredients were detectable on the vegetation surrounding recently planted areas, although at very low concentration levels (ng/g). Based on these findings, it was proposed that the contamination of the air and surrounding environment was the result of the abrasion and separation of the insecticide coating away from seed kernels during planting, and the subsequent expulsion of insecticide particles into the environment via the exhaust fan system of the sowing machine. This discovery forms the basis for the now widely accepted mechanism of pesticide drift from neonicotinoid-treated seeds. Indeed, more recent work has further demonstrated that the sowing of treated seeds results in the development of a “toxic” dust cloud around the planting machine, where concentrations of insecticide particles reach levels of up to 30 μg/m^3^, a concentration sufficient to kill bees passing through in a single flight (Girolami et al. 2012, 2013). In contrast, water droplets (both guttations and dew) collected from exposed vegetation adjacent to sown areas would not present acute risk of toxicity to bees (Marzaro et al. 2011).

#### Developments

It is now known that the dissemination of neonicotinoid-contaminated dust is exacerbated by the addition of seed lubricants during planting. In North America, for instance, talc, graphite, or a combination of these minerals in a finely powdered form is typically mixed with seeds to minimize friction and ensure smooth seed flow during planting (Krupke et al. 2012). Lubricants are added directly into the planter with pesticide-treated seeds; inevitably some amount of lubricant powder fails to adhere to seeds during the sowing process. This residual lubricant remains behind in the planter to be exhausted, either immediately (i.e., during seed sowing) or later during routine cleaning of planting equipment. Because this powder comes into direct contact with treated seeds, it can act as a carrier of abraded seed coating. In fact, residual talc lubricant has been shown to contain high concentrations of seed treatment compounds, including the protectant fungicides metalaxyl and trifloxystrobin, and up to 15,000 μg/g of neonicotinoid active ingredients (Krupke et al. 2012), a concentration several orders of magnitude above the contact lethal dose for honeybees.

Neonicotinoid-contaminated dust poses a risk to nontarget organisms through a variety of mechanisms. For instance, abraded insecticide particles that settle on surrounding vegetation can contaminate flowering plants (including insect-pollinated crops, cover crops, and weeds), and thus provide a means of exposure for pollinators utilizing these floral resources (Greatti et al. 2003). In fact, residues of the neonicotinoid clothianidin have been detected (up to 9 ng/g) on dandelions, a key early season resource for honeybees, following the planting of clothianidin-treated maize (Krupke et al. 2012). Exposure to contaminated dust could pose risks for nontarget organisms whether they are exposed to insecticides by contact (dust cloud or deposition on vegetation) or through the ingestion of contaminated plant products (pollen, nectar, etc.). Indeed, high concentrations (above 20 ng/g) of seed treatment pesticides (clothianidin and thiamethoxam) have been detected in samples of stored pollen taken from colonies experiencing losses during corn planting in the USA (Krupke et al. 2012). It is important to note that the reported pesticide concentrations from the flowers and nectar of seed-treated crops are below levels that would induce acute toxicity in honeybees foraging in recently planted areas. Therefore, this exposure mechanism is unlikely to explain the high incidence of bee deaths during the seed planting period. However, a possibly complementary exposure route for nontarget organisms during the planting period is via direct contact with contaminated dust in-flight (e.g., during pollinator foraging flights that pass through areas being sown with treated seeds). In-flight exposure could be of special consequence for organisms like honeybees that possess abundant pubescence on their body surface. This pubescence renders bees more likely to accumulate and retain small particles dispersing in the air, and furthermore creates electrostatic-friction with the air which can enhance the attraction of small particles by bees (Vaknin et al. 2000). By conditioning honeybees to fly through planter-generated dust clouds, Girolami et al. (2012) and Tapparo et al. (2012) unequivocally demonstrated that honeybee foragers can acquire lethal doses of neonicotinoid residues in-flight, with concentrations ranging from 50–1,200 ng/bee (Girolami et al. 2012; Tapparo et al. 2012). The latter value of 1,200 ng/bee is 60 times the lethal dose of 20 ng/bee (US EPA 1993). As such, exposure to pesticide residues at the concentrations documented by Tapparo et al. (2012) would undoubtedly elicit acute toxicity in honeybees, and furthermore this in-flight mechanism of exposure to contaminated dust could explain the observations of dead and dying bees during the planting of neonicotinoid-treated seeds in various jurisdictions worldwide. Moreover, the sheer magnitude and frequency of crop treatment with neonicotinoid insecticides (e.g., the majority of maize, soybeans, wheat, and rapeseed), combined with the coincidence of seed sowing and the flush of spring blossoms may create scenarios where the flight paths of bees are likely to overlap, both in time and space, with planting activities in many areas. As a result, bees may be at greater risk of in-flight exposure to lethal doses of insecticides in planter exhaust as they forage near agricultural areas that increasingly dominate many landscapes.

Given the widespread risks posed to pollinators, efforts have been made to mitigate the dispersion of contaminated dust in recent years. These include modifications to planting equipment using a variety of devices (collectively known as “deflectors”) that direct seed dust down into the seed furrow before it is closed, as well as improvements to the quality of seed treatment formulations. Although these measures have the potential to reduce dust movement away from the planter (Nikolakis et al. 2009; Balsari et al. 2013), field experiments suggest that neither alterations to seed coating quality nor modifications to drilling machines eliminate the incidence of honeybee deaths during the sowing of treated seeds (Girolami et al. 2012, 2013; Tapparo et al. 2012). In addition, modifying equipment by adding deflectors can be laborious, time consuming, and potentially counter-productive if these changes affect the accuracy and precision of seed placement (Pochi et al. 2012). Taken together, these factors make this option less appealing to growers and planter manufacturers alike. Furthermore, because the seed lubricants used in North American planting equipment (talc and graphite) have been found to abrade pesticides from the seed coat during planting, efforts have been made to transition to less abrasive lubricants. Bayer CropSciences has recently developed a novel lubricant powder to reduce the development of dust during the sowing of treated seeds. This powder, known as “fluency agent” has been tested in North American production fields, but there are currently no published data regarding planting efficacy and/or dust reduction. However, in acknowledging that most incidents of acute honeybee poisonings in recent years were the result of contact with planter dust, the Canadian Pest Management Regulatory Authority (PMRA) recently specified that all treated corn and soybean seed must be sown using “fluency agent”, beginning in 2014 (PMRA 2013). The European Food Safety Authority (EFSA) has recently acknowledged that bees can be directly contaminated by poisoned dust around the drilling machine during seed sowing (EFSA 2013a, b, c, d). Similarly, the United States Environmental Protection Agency (EPA) has highlighted planter dust as an area of concern and a relevant exposure route in a recent white paper proposing a risk assessment for pollinators (US EPA 2013).

#### Conclusions

The relative importance of contaminated planter dust containing neonicotinoids and other seed treatment pesticides and its corresponding impacts on the health of honeybees and other nontarget organisms has been debated since these products were first registered for use (Schnier et al. 2003). While it is now generally accepted that honeybees encountering contaminated dust will experience mortality events, recent overviews of seed treatments and their impacts on honeybee health differ in the degree of importance they assign to this source of pesticide exposure (Cresswell 2011; Goulson 2013; Nuyttens et al. 2013). While the impacts of contaminated planter dust have been studied closely for managed pollinators like honeybees, this area remains largely unexplored in the case of other pollinators, particularly solitary species, and species with small foraging radii. The degree to which the dispersion of contaminated dust affects nontarget lands, waterways, and the organisms living there in both the short- and long-term is currently unclear; however, given the millions of hectares of treated seed planted annually worldwide, neonicotinoid-contaminated dust stands out as a key route of pesticide exposure for nontarget organisms.

### Soil—environmental fate and exposure of neonicotinoid insecticides in soils

#### Introduction

As outlined above, the primary method for application of the systemic neonicotinoids and fipronil for agricultural pest control is the planting of seeds that are coated with the insecticide. For other pest control uses, insecticides can be applied directly to soils for uptake by plants or to the plants themselves by stem injections (Tattar et al. 1998; Kreutzweiser et al. 2009). The subsequent breakdown of plant material containing insecticide residues can release concentrations back into the soils, thereby providing a further route of soil contamination (Horwood 2007).

Neonicotinoid and fipronil insecticides have been shown to pose a risk of harm to earthworms and other soil invertebrates (Pisa et al. 2014, this issue). In doing so, they have the potential to adversely affect soil ecosystem services (Chagnon et al. 2014, this issue). Therefore, an understanding of the fate and dynamics of insecticide residues in soils is necessary for an environmental risk assessment. Below, we review the literature on the fate of neonicotinoids in soils.

#### Temporal dynamics

Neonicotinoids are applied directly to the soil or are released from seed coatings into the soil where they are available to be taken up by plant roots and incorporated into plant tissues (Mullins 1993). Plant uptake processes together with natural degradation of these pesticides is believed to cause soil concentrations to rapidly decrease over time (Horwood 2007). For example, in a field experiment, imidacloprid concentration declined from 652 μg/kg 30 days after seeding to 11 μg/kg by the time of harvest (130 days after seeding), by which time it was not significantly higher than in untreated soils (5 μg/kg) (Donnarumma et al. 2011). Natural degradation was also reported for several insecticides, including imidacloprid and fipronil used to fight termites in Australia with 95 % loss measured after 1 year in situ at one site and 50 % at another site (Horwood 2007).

Nevertheless, neonicotinoids can remain present in measurable concentrations for long periods (months to years) in the soil. Bonmatin et al. (2005a) analyzed the concentration of imidacloprid in 74 soils covering a broad range of climates, soil type, and agricultural practices in France. Imidacloprid was detected in 91 % of the samples (>0.1 μg/kg), although only 15 % of the sites had been planted with treated seeds during the same year. Imidacloprid could be detected in 100 % of the soils seeded with treated seeds in the same year. Imidacloprid was detected in 97 % of soils seeded with treated seed 1 or 2 years before the study. Interestingly, the concentrations were higher in the soils that had been treated consecutively during 2 years before the analysis than in those that received treated seed only 1 year before the analysis (Bonmatin et al. 2005a), indicating that imidacloprid can accumulate over time in soils. These observations are in line with others who have reported a long persistence of neonicotinoids in the environment (Fossen 2006; Gupta and Gajbhiye 2007). In contrast, Bonmatin et al. (2005a) found no detectable residues of neonicotinoids in soils of agricultural fields under organic farming practices.

#### Half-life—ranges (soil)

Degradation of neonicotinoids and fipronil in soils depends on factors such as soil type (especially texture and organic matter content), ultraviolet radiation (for surface degradation), moisture, temperature, and pH and will therefore vary from place to place. In the mid and higher latitudes, the half-life will be longer than in tropical regions because of fewer sun hours, lower sun light intensity, and lower temperatures.

Calculated half-lives of imidacloprid in soil range over 1 order of magnitude from 100 to 1,230 days following application (Baskaran et al. 1999). The shortest recorded half-life of imidacloprid in the field is 107 days in turf-covered soils in the humid subtropical climate of Georgia, USA (Cox 2001), while according to Belzunces and Tasei (1997), the half-life of imidacloprid ranges between 188 and 249 days. However, ranges of 27 to 229 days, 997–1,136 days (in laboratory studies) (Scorza et al. 2004; Fossen 2006), 455–518 days (Fernandez-Bayo et al. 2009), 28–46 days (in India) (Sarkar et al. 2001), and even 1,000 days in soil and bedding material (Baskaran et al. 1999) have been reported. The half-life for imidacloprid in soils of seed-treated fields was about 270 days in France (Bonmatin et al. 2005a). However, no decrease in concentration was observed over a 1-year period following treatment in a field test in Minnesota (Cox 2001). Half-life of imidacloprid ranged from 3 to 4 months to over 1 year in soils in the USA (US EPA 1993a) and was longer under higher pH conditions (Sarkar et al. 2001). Based on data in Anon (2006), Goulson (2013) calculated the half-life of 1,250 days for loam in the UK.

The calculated half-life of clothianidin in soil varies even more than that of imidacloprid and ranges between 148 and ca. 7,000 days (DeCant 2010). However, degradation is higher at soil surfaces owing to UV degradation (Gupta et al. 2008a). Goulson (2013) reviewed estimated DT50 (half-life) in soil for the other neonicotinoids as well and reported 31–450 days for acetamiprid, 75–82 days for dinotefuran, 8 days for nitenpyram, 3.4–>1,000 days for thiacloprid, and 7–335 days for thiamthoxam.

For fipronil, half-life times in soil range between 122 and 128 days in lab studies (sandy loam). In field studies, the half-life time ranges from 3 to 7.3 months (US EPA 1996) although a half-life 24 days was reported in a cotton field experiment (Gunasekara et al. 2007; Chopra et al. 2011).

#### Effect of water content (soil)

Although these half-life ranges seem very broad, they can be explained to some extent by environmental conditions. Acetamiprid half-life is known to depend strongly on soil conditions, being almost 10 times longer under dry conditions (150.5 and 125.4 days for air-dried soils for 1 and 10 μg/g dosage, respectively) than at field capacity moisture (17.4 and 15.7 days) and submerged conditions (19.2 and 29.8 days) (Gupta and Gajbhiye 2007). Similar results were obtained in lab studies for thiamethoxam, with half-life increasing from submerged conditions to field capacity and to dry conditions (46.3–75.3, 91.2–94.1, and 200.7–301 days, respectively) (Gupta et al. 2008b).

Similarly, fipronil half-life in Australian Red Earth loam soils increased from 68 days at 60 % maximum water-holding capacity (MWHC) to 198 days when the moisture content was 15 % MWHC. By contrast, no significant difference was observed between MWHC of 90 and 165 % (Ying and Kookana 2006).

These results suggest that degradation is related to microbial activity, which is strongly reduced in dry soil conditions and somewhat reduced in saturated soil conditions as a result of low oxygen. In addition, lower concentrations in soils of higher water content may also be due to dilution effects. The concentrations of other chemical compounds in the soil are known to vary in relation to soil moisture content (Misra and Tyler 1999), and this is likely also true for neonicotinoids, but to our knowledge not studied directly. Such changes in concentrations of solutes can in turn affect soil organisms and the concentrations of pesticides in guttation fluid from vascular plants. In support for this view, thiamethoxam concentrations in guttation liquid collected from corn plants were indeed shown to be higher in low soil moisture conditions than in high soil moisture conditions (Tapparo et al. 2011).

#### Dose dependency of decay

Decay of pesticides has been shown to depend on the dose applied. We did not find any studies on this topic for neonicotinoids, but, in the case of fipronil, dissipation was shown to be rapid (24 days) at relatively low dose (56–112 g active ingredient/ha) (Chopra et al. 2011). Fipronil was also found to exhibit a dose-dependent rate of decay within a similar range (0.15, 0.75 and 3.0 g active ingredient/m^2^) in Australian Red Earth loam soils (Ying and Kookana 2006). The time for 50 % loss of active ingredients to occur increased approximately fourfold from low to high application rates (145–166 days at lowest rate to 514–613 days at highest rate). Although we did not find published reports of dose-dependent decay among neonicotinoid insecticides, we raise this as a possible further factor affecting concentrations in soils.

#### Effect of temperature on decay

Imidacloprid degradation was temperature-dependent in a lab incubation experiment (clay soil). Half-lives decreased from 547 to 153 days and finally to 85 days at incubation temperatures of 5, 15, and 25 °C, respectively (Scorza et al. 2004). The same authors report results from a field experiment in which imidacloprid concentrations declined rapidly at first (50 % between May and September) but then no significant change could be detected during the cold months of the year, suggesting a temperature effect (Scorza et al. 2004). High temperature (experimental site in Hisar, 100 km NW of new New Deli, India) was shown to increase the degradation of fipronil (Chopra et al. 2011).

#### Leaching and other causes of concentration changes

Independently from uptake by plants or microbial breakdown, concentrations of neonicotinoids and fipronil may change owing to movement in the soil. Two main factors determine such movements: (1) the concentration or identity of dissolved molecules in the soil solution and (2) the sorption on soil particles. Neonicotinoids are mobile in the soil and thus represent a potential contamination threat to surface water and groundwater.

Leaching of pesticides is one of the main mechanisms responsible for the contamination of groundwater and surface water. The leaching process is highly variable across different soil types, pesticide formulations, and application methods (Gupta et al. 2002; Huseth and Groves 2014). The presence of cracks or other macropores in the soil (earthworm burrows, root channels, etc.), or less-structured soil can lead to preferential flows that bypass the most chemically and biologically reactive topsoil, thus facilitating the high mobility of pesticides (Scorza et al. 2004).

One way of determining the leaching potential of a substance is by calculating the Groundwater Ubiquity Score (GUS). It is calculated from the sorption coefficient (*K*_oc_) and the soil halftime (DT50) in the following manner (Gustafson 1989):

$$ \mathrm{GUS}={ \log}_{10}\left(\mathrm{DT}50\right)\times \left(4-{ \log}_{10}\left({K}_{\mathrm{oc}}\right)\right) $$

As seen in Table 1 and according to GUS, dinotefuran and clothianidin have a very high leaching potential, imidacloprid and thiamethoxam have a high leaching potential, while fipronil and nitenpyram are classified as possible leachers (PPDB 2012). Contrary to the other systemic pesticides, acetamiprid and thiacloprid break down readily in soil, thereby decreasing the risk of leaching. But the most commonly used agricultural neonicotinoids (imidacloprid, clothianidin, and thiamethoxam) each have a GUS leaching potential index greater than 3.7.

Imidacloprid is known to leach more rapidly through soil columns than other tested pesticides, including common water contaminants such as the organophosphate insecticides chlorpyrifos and diazinon and the herbicide diuron (Vollner and Klotz 1997; Cox 2001). Comparative modeling conducted by the US EPA have shown that imidacloprid had the highest leaching potential among 14 turf insecticides (US EPA 1993b). This high mobility was also confirmed in a field experiment in which imidacloprid was shown to be very mobile in irrigated soil (Felsot et al. 1998). This is also the case for greenhouse soil; Gonzalez-Pradas et al. (2002) report that imidacloprid penetrates the first 40 cm of soil within 2 years of the first application in greenhouses. Gupta et al. (2002) investigated the leaching behavior of different imidacloprid formulations and found that imidacloprid recovery in 25 cm column leachate varied between 28.7 (analytical grade) and 44.3 % (water-dispersible powder). The heightened leaching potential in commercially available formulations is attributed to the surfactants that were added to the product. Indirect evidence of leaching is also shown by a nearly 50 % drop of imidacloprid concentration (120 vs. 220 ppb) in Hemlock tissue when applied to soil in autumn versus spring (Cowles et al. 2006). Thiamethoxam was also shown to be highly mobile in soil. In a soil column leaching experiment, the equivalent of 65 cm of rainfall caused leaching of 66–79 % of the applied thiamethoxam and no residues could be detected in the soil (Gupta et al. 2008b). These results clearly show that neonicotinoids have a high potential to leach vertically down the soil profile or laterally through soil flow paths and contaminate surface and groundwater.

Mobility of fipronil and of its metabolites (desulfinyl, sulfide, and sulfone derivatives) was observed down to 15 cm, but only traces were found at higher depths (15–30 cm) in three Australian Red Earth loam soils (sandy, loamy, and clay) overlain by 5 cm of quartzite sand. However, experimental plots were covered by plastic liners and fiber cement during the course of the experiment, thus limiting the leaching due to rain (Ying and Kookana 2006). The same authors reported an experiment on two repacked soils (sandy loamy and clay, respectively) with alternative wet-dry weekly cycles (7 days dry followed by 20 mm of rain). Fipronil was added at a high concentration (3 g/m^2^ active ingredient, which in a parallel experiment was shown to result in longest half-life), and bromide was used as a tracer. Mobility was minimal in both soils and not related to the behavior of bromide (highly leached in the sandy loamy soil but not in the clay soil) (Ying and Kookana 2006). Limited fipronil mobility was also demonstrated in Australian soils despite rather dry conditions: although measured annual rainfall was only 432.1 mm, mostly falling during the second half of the experiment, significant downward movement of fipronil was measured (Ying and Kookana 2006). Fipronil was found to bind to soil organic matter, increasing in the range 0.1–6.5 % (Bobé et al. 1997; Gunasekara et al. 2007) and this may explain the low bioaccumulation measured in fungi grown on compost with different concentration of fipronil (Carvalho et al. 2014).

#### Conclusions

Neonicotinoid and fipronil concentrations in soils typically decline rapidly after application, by hydrolytic, photolytic, and microbial degradation, by plant uptake, by sorption to soil particles, and by leaching to receiving waters. However, in some soil conditions, neonicotinoid and fipronil concentrations can persist, and possibly accumulate, for months or years. Persistence is highest under cool, dry conditions and, at least for neonicotinoids but possibly also for fipronil, in soils with high organic matter content. Given that neonicotinoids and fipronil are widely used in agricultural settings and can persist in drier, organic-enriched soils, which are common in agricultural fields, their residues in agricultural soils may pose a risk to soil organisms (Pisa et al. 2014, this issue). The uptake of soil-borne residues by plants expands this risk of exposure to other nontarget organisms such as those feeding on living or decomposing plant material, and those collecting nectar and pollen, although little is known about biologically-relevant concentrations found in nontarget plants and the effects of these concentrations upon other organisms.

While the environmental fate of neonicotinoids and fipronil in soils has been examined in several field and laboratory studies, some uncertainties remain. It is not always clear to what process the half-lives correspond. Half-life values are clear for imidacloprid hydrolysis (33 to 44 days at pH 7 and 25 °C) and photolysis (under 3 h) (Fossen 2006), but the term “half-life” is also used when discussing decreasing concentrations over time in soil regardless of the mechanism. For example, Cox writes “*The shortest half-life (the amount of time required for half of an applied pesticide to break down or move away from the test site) was 107 days in turf-covered soil in Georgia*.” (Cox 2001). There are several possible ways by which pesticide concentrations in soils can decrease including uptake by plants, leaching through the soil profile (a demonstrated important process), lateral drainage (in cases of sloping terrain), abiotic or biotic degradation, evaporation (although unlikely given to the low volatility of at least imidacloprid (Fossen 2006)), and dilution (if soil moisture content increases between measurements).

Although some of the mechanisms of dissipation or breakdown have been shown for parent compounds, little is known about the concentrations and dynamics of neonicotinoid and fipronil degradation products and metabolites. Progress on characterizing and tracking metabolites in soils is impeded by the lack of sensitive analytical methodology, and by the fact that information on the chemical structure of metabolites and the availability of reference materials is often proprietary and not available to researchers. Early indications from unpublished studies on metabolites of imidacloprid suggest that several metabolites can be found and they can be more toxic to invertebrates than the parent compound (Suchail et al. 2001; Simon-Delso et al. 2014, this issue).

### Water—environmental fate and exposure of neonicotinoid and fipronil insecticides in water and sediments

#### Introduction

The contamination of surface water with pesticides is an ongoing concern worldwide. Innovations in pesticide composition and application methods present new solutions as well as challenges. The invention of neonicotinoids and fipronil heralded a new era of pest management, with a higher versatility in application methods and a high target specificity for invertebrates (Jeschke and Nauen 2008). However, these new pesticides present their own set of problems. There are numerous ways for systemic pesticides such as neonicotinoids and fipronil to contaminate groundwater or surface water. The increasing use of these compounds worldwide therefore raises concerns about higher and more widespread contamination of aqueous environments (Overmyer et al. 2005; Tišler et al. 2009). In addition to toxicity, pesticide persistence, metabolite characteristics, the source of contamination and level of exposure are all important for determining the impact of these compounds on aquatic organisms and ecosystems. The persistence of systemic pesticides in the aqueous environment varies with field conditions. These include exposure to sunlight, pH, temperature, the composition of the microbial community, and also the formulation and quantity of the pesticide.

##### Photodegradation

When studied under laboratory conditions, photolysis plays a major role in degradation of systemic pesticides in water (Table 1). Imidacloprid undergoes photolytic degradation rapidly (CCME 2007). However, it proves difficult to find consistent data. Tišler et al. (2009), for example, stored analytical-grade imidacloprid in distilled water (varying concentrations, 8.75–140 mg/L) in the dark at cold temperatures (3 ± 2 °C) and in room light at 21 ± 1 °C. The samples stored in the cold temperature showed no variation during 22 days, while the samples stored at room temperature showed decreasing levels of imidacloprid during this period, dependent on the initial concentration. The higher concentrations (105 and 140 mg/L) decreased by up to 24 % in this period, while levels of 70 mg/L and lower stayed the same. Although the authors hypothesize that this can be attributed to photolytic breakdown in light, the large temperature difference between the two methods is not taken into account in this statement.

In the absence of light, the DT50 of neonicotinoids and fipronil in sediments varies considerably. Thiacloprid is reported to have the shortest DT50, 28 days, while imidacloprid persists the longest at 130 days (PPDB 2012). This last finding on imidacloprid is confirmed by Spiteller (1993) and Krohn and Hellpointner (2002), and cited in Tišler et al. (2009), who found DT50 values of 130 and 160 days for different types of sediments.

##### Temperature

The rate of hydrolysis of imidacloprid increases with temperature (Zheng and Liu 1999; Scorza et al. 2004). The first authors reported an effect of temperature on half-life times of imidacloprid in soil for example (547 days at 5 °C to 89 days at 25 °C).

##### pH

The degradation rates of neonicotinoids and fipronil in water also vary with pH. PPDB (2012) and US EPA (2005) reports that imidacloprid is stable at a pH between 5 and 7, while the half-life time at pH 9 is about 1 year at 25 °C, thereby indicating a decreasing DT50 with increasing pH. Thuyet et al. (2013) studied degradation of imidacloprid and fipronil at pH levels relevant for rice paddies. Kept at 18.2 ± 0.4 °C and in the dark, the initial concentrations of 60 and 3 μg/L, respectively, for analytical-grade imidacloprid and fipronil, were based on field-realistic concentrations found in paddy fields after application of these pesticides. After an initial decrease in concentration on the first 7 days, the concentration of imidacloprid remained stable at pH 7, but continued to decrease at pH 10. The authors estimated a DT50 of 182 and 44.7 days for imidacloprid at pH 7 and 10. However, Sarkar et al. (1999) found an average half-life of 36.2 days at pH 4, which increased to 41.6 days at pH 9. It should be noted that these results were obtained with commercial formulations (Confidor and Gaucho) at an ambient temperature of 30 ± 5 °C, which is a very wide range. The relatively high temperature will increase the degradation rate, making these results difficult to translate to the majority of field conditions.

Guzsvány et al. (2006) studied the effect of pH on degradation of four different neonicotinoids (at 23 °C) and found that imidacloprid and thiamethoxam degraded more rapidly in alkaline media, while staying relatively stable at pH 7 and 4. Likewise, fipronil degradation is strongly pH dependant, with hydrolysis half-life declining from >100 days at pH 5.5 and 7 to 2.4 h at pH 12 (Bobé et al. 1997). In contrast, acetamiprid and thiacloprid degraded more rapidly in acidic conditions while remaining stable for about 30 days in alkaline conditions. In contrast, several sources indicate that imidacloprid more readily degrades under alkaline conditions (Zheng and Liu 1999; US EPA 2005 in CCME 2007). An experiment determined that, while no hydrolysis products were detected at pH 5 and 7 at any sampling intervals, imidacloprid transformed slightly at pH 9, with a calculated half-life of 346.5 days (Yoshida 1989 report in CCME 2007). Based on these results, the compound is stable to hydrolysis at environmentally relevant pH (CCME 2007).

##### Field conditions

Although most neonicotinoids and fipronil degrade in sunlight, in field conditions, the proportion of transmitted sunlight in water depends on water depth, turbidity, and the wavelength of the incident radiation (Peña et al. 2011). Overall, degradation under field conditions results in variable concentrations through time. In a field experiment, Sanchez-Bayo and Goka (2006) observed an initial decrease of imidacloprid in rice paddies with a starting concentration of 240 μg/L, but the concentration stabilized at 0.75 μg/L for the entire 4-month duration of the experiment. Kreutzweiser et al. (2007) report a declining rate of degradation over time for imidacloprid (initial doses, 0.001–15.4 mg/L) in water of laboratory microcosms, with a dissipation of about 50–60 % after 14 days for the higher doses. The authors conclude that aqueous imidacloprid concentrations could therefore persist in natural water bodies for several weeks at measurable concentrations. Others have reported surface water concentrations of imidacloprid that persist under field conditions (Van Dijk et al. 2013; Main et al. 2014). However, in a study to aid registration of imidacloprid as a potential control measure for burrowing shrimp, imidacloprid was applied to tidal mudflats in Willapa Bay, USA, in three application rates (0.28, 0.56, and 1.12 a.i./ha). After 28 days, imidacloprid was still detectable in the sediment (limits of detection (LOD) of 2.5 ng/g). However, it dissipated very quickly from the water, being detectable only in one of the three test blocks the day after application. This was attributed to the fast dilution and low sorption potential of imidacloprid (Felsot and Ruppert 2002).

In urban areas, most pesticide runoff is collected in a sewage system and will often undergo treatment at a wastewater plant before being returned to the surface water. Although degradation of thiamethoxam does take place in wastewater, with a half-life of 25 days while in the dark, this is not the case for all neonicotinoids. For example, thiacloprid concentrations in wastewater remained stable whether exposed to sunlight or not, over a 41-day period (Peña et al. 2011). Imidacloprid has also been detected in wastewater treatment plants in Spain (Masiá et al. 2013).

Despite laboratory studies suggesting that clothianidin is susceptible to rapid degradation or dissipation through photolysis (aqueous photolysis DT50 < 1 day), the slow rate of dissipation in field conditions indicates that photolysis in natural systems does not play a large role in the degradation process (US EPA 2010). Peña et al. (2011) demonstrated the susceptibility of thiamethoxam to direct photolysis, but found clothianidine and thiacloprid to be stable under direct sunlight. Clothianidin is reported to be stable under environmentally realistic pH and temperatures (US EPA 2010).

##### Metabolites

Degradation of neonicotinoids often produces secondary metabolites in water, some of which have been proven to have an equal or greater toxicity than their parent compounds (Suchail et al. 2001). An example is clothianidin, a metabolite of thiamethoxam, which is itself commercially available as an insecticide. For an overview, see Simon-Delso et al. (2014, this issue).

#### Sources of contamination in water

**Fig. 1:**
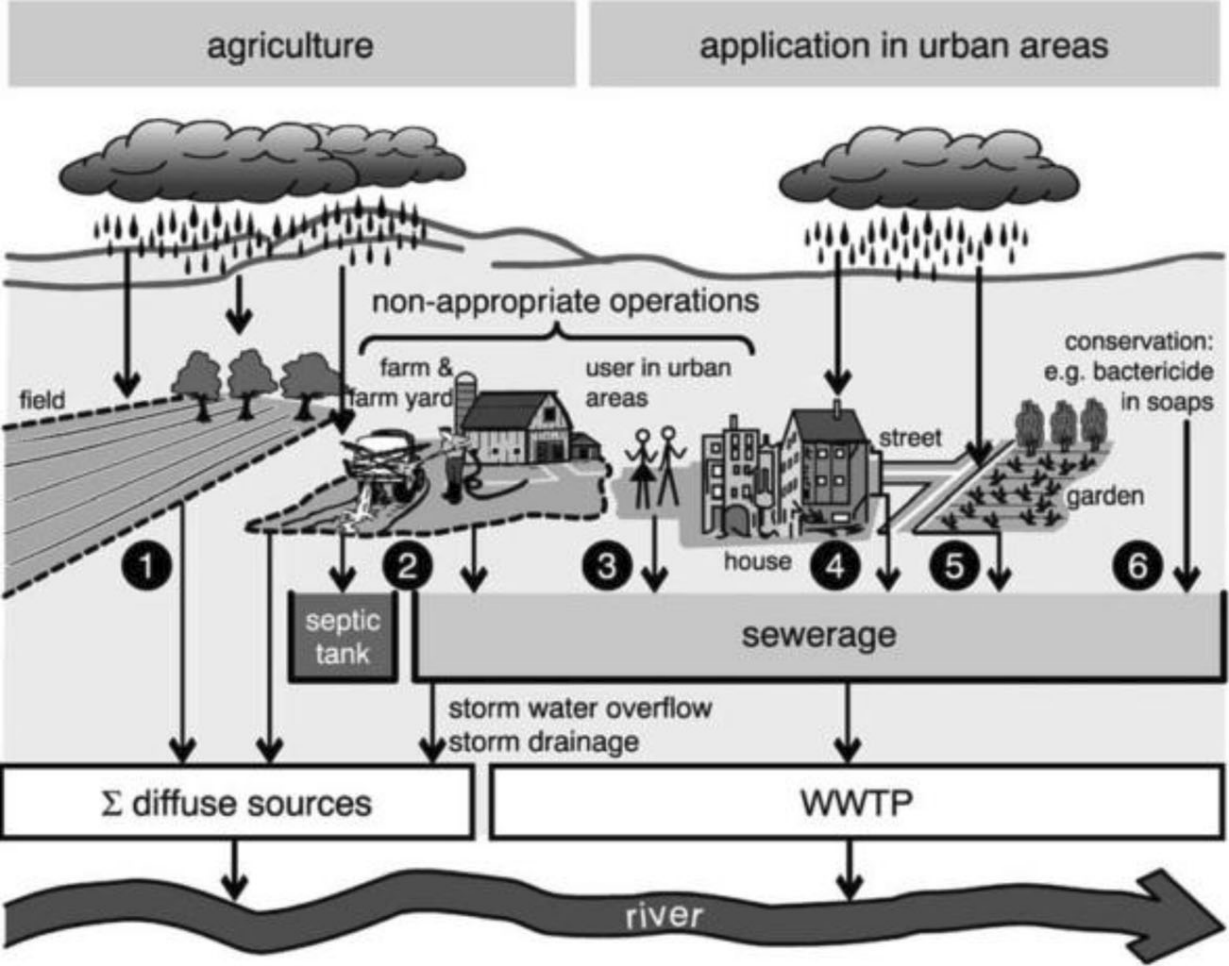
Important applications and major pathways for pesticide transport into surface waters. *1* Field—spray and dust drift during application, surface runoff, and leaching with subsequent transport through drainage channels during rain events. *2* Farm and farmyard—improper operations (e.g., filling of sprayers, washing of measuring utilities, disposing of packing material, driving with seeping sprayers, and cleaning of spraying equipment). These operations are done either at locations, which are drained to the sewerage, to the septic tank or into surface waters. *3* Like 2 for pesticide users in urban areas. *4* Pesticides in building material—leaching during rain events. *5* Applications on lawns, streets, and road embankments—runoff during rain events. *6* Protection of materials—e.g., products containing antifouling ingredients that get into the sewerage (e.g., detergents and cosmetics) (source, Gerecke et al. 2002)

Systemic pesticides used on agricultural fields, grass, turf, or hard surfaces such as lawns, golf courses, or concrete may contaminate surface and/or groundwater through (foliar) runoff, as well as through leaching, (subsurface) drains, spillage, greenhouse wastewater, and spray or dust drift (Gerecke et al. 2002). In addition, water on the soil surface of treated fields, temporary pondage, may contain high concentrations of systemic pesticides (Main et al. 2014). In sporadic events, flooding of greenhouses and the subsequent emptying thereof into surface water may result in severe contamination locally. In addition, when applied as stem injection to trees, the falling leaves in autumn may provide a source of contamination to water bodies (Kreutzweiser et al. 2007). Figure 1 provides an overview.

##### Spray or dust drift

Spray application may lead to direct contamination of surface water. This may be caused by unintentional overspray, careless application, or wind dispersal. In addition, dust emission from treated seeds during planting has the potential to drift to adjacent areas. EFSA (2013b, f) gives the percentage of dust drift deposition on the surrounding vegetation from 0.01 % in sugar beet to 7.0 % for maize. Although surface water does not have the three-dimensional catchment properties of surrounding vegetation, it still indicates that measureable amounts of these pesticides may potentially contaminate surface water directly through drift. For example, Tapparo et al. (2012) carried out particulate matter emission tests with different types of commercially available treated maize seeds. While the exact distance that the dust travels depends on atmospheric conditions, it is reasonable to assume that such particulate matter can drift to nearby surface water.

##### Runoff

Neonicotinoids and fipronil are often used to control insect pests in urban or residential areas. Use of these insecticides on ornamental plants or near impervious surfaces creates a potential mode of contamination for aquatic ecosystems through runoff during rainfall or irrigation (Armbrust and Peeler 2002; Haith 2010; Thuyet et al. 2012). Runoff may include dissolved, suspended particulate and sediment-adsorbed pesticides (van der Werf 1996). Imidacloprid and fipronil runoff from turf and concrete surfaces was studied by Thuyet et al. (2012). During their experiment, they subjected turf and concrete surfaces to simulated rainfall at different points in time and with different treatments (turf, granular imidacloprid; concrete, emulsifiable concentrate of imidacloprid and suspension concentrate of fipronil). Their findings indicate a high runoff of imidacloprid on concrete surfaces following 1.5 h after application, with peaks up to 3,267.8 μg/L, 57.3 % of the amount applied. However, percentages dropped between 1.0 and 5.9 % 1 day after the application. No imidacloprid was detected in runoff 7 days after application. Mass losses of fipronil from concrete surface runoff were comparable to imidacloprid with 0.9 to 5.8 %. However, the concentration of toxic byproducts from fipronil runoff was high in all samples. The findings on turf surfaces for imidacloprid varied largely between repeated samples, with between 2.4 and 6.3 % of applied mass product detected in the runoff.

Runoff of these pesticides can also occur in agricultural settings. Residues can occur on plant surfaces after foliar applications or accumulation of pesticide-contaminated dust, and these residues can be washed off during rain events leading to contamination of surface waters. Climate change is expected to play a role in altering pesticide environmental fate in the future. The likelihood of runoff increases with precipitation levels, with increased frequency and intensity of storm events and with increasing pest pressure under climate change effects. As a consequence, the risk of pesticide runoff is likely to be elevated (Kattwinkel et al. 2011). Bloomfield et al. (2006) examined the impacts of this for pesticide behavior in groundwater and surface water in the UK. Pesticide mobility is expected to increase through more frequent heavy rainfall events, increased soil erosion, and cracking of soils leading to faster by-pass flows in winter. In the drier periods, lower flow in rivers also has the potential to increase pesticide concentration and accumulation in sediments (Masiá et al. 2013). On the other hand, higher soil and surface water temperatures due to climate change will decrease some pesticide half-life times. While the overall impact is difficult to predict, increased transport to surface and groundwater of soluble substances such as several neonicotinoids seems likely. For clothianidin, for example, increased mobility is expected, but not the predicted decrease in half-life time as clothianidin is not sensitive to temperature changes. The future increased potential of such pesticides to reach and accumulate in surface and groundwater is an aspect that requires attention and warrants further research. Similarly, increases in the risk of flooding, especially in greenhouses, could result in washing out of systemic pesticides to the environment (Blom et al. 2008).

##### Drainage

Systemic pesticides are also used in greenhouses, where application techniques include drenching of flower bulbs or chemigation (adding chemicals to irrigation water). The wastewater drainage from these greenhouses is often released into surface water and contains high levels of neonicotinoids. Kreuger et al. (2010) studied pesticides in surface water next to vegetable crops and greenhouses in different regions in Sweden. The authors found imidacloprid present in 36 % of the samples, including all samples taken from stream water draining areas with greenhouse cultivation. The highest concentration of imidacloprid was 9.6 μg/L, substantially higher than in other areas with outdoor cultivation of vegetables. Acetamiprid and thiametoxam were also detected, in 9 and 3 % of the samples, respectively. Only a trace of thiacloprid was found once.

#### Exposure

##### Environmental concentrations

Contamination of surface water with neonicotinoids or fipronil has been reported in various countries as early as the 1990s. In the Netherlands, imidacloprid was one of the top three of the substances exceeding the ecotoxicological limit (13 ng/L) since 2004, and has been shown to occur in surface water at up to 25,000 times that amount (Van Dijk et al. 2013). In 2010 and 2011, 75 surface water samples were taken from agricultural regions in California. Imidacloprid was detected in 89 % of the samples and the US EPA toxicity benchmark of 1.05 μg/L was exceeded in 19 % of the samples (Starner and Goh 2012). In a more recent study, Main et al. (2014) surveyed levels of neonicotinoids in water and sediment in the Canadian Prairie Pothole Region. A total of 440 samples were taken before seeding (2012 and again in 2013), during the growing season (2012) and after the harvest of crops in fall (2012). At least one of the following neonicotinoids, clothianidin, thiamethoxam, imidacloprid, or acetamiprid was found in 16 to 91 % of the samples, depending on the time of sampling. Clothianidin was the most commonly detected chemical of the group during three of the four sampling periods, while thiamethoxam was predominant in water samples during the fourth sampling period (after harvest 2012). Maximum concentrations detected in the water were 256 ng/L for imidacloprid (mean, 15.9 ng/L; wheat crops after seeding 2012), 1,490 ng/L for thiamethoxam (mean, 40.3 ng/L; canola after seeding 2012), 3,110 ng/L for clothianidin (mean, 142 ng/L; canola after seeding 2012), and 54.4 ng/L for acetamiprid (mean, 1.1 ng/L; canola after seeding 2012).

Concentrations in soil water exceeding 20 times the permitted level in groundwater (EU directive at the time of the study 1997–1999, i.e., 91/414) were measured in greenhouse soil in Almeria, Spain (Gonzalez-Pradas et al. 2002). A large-scale study of the Guadalquivir River Basin in Spain by Masiá et al. (2013) detected imidacloprid in 58 % (2010) and 17 % (2011) of the samples, with concentrations in these 2 years ranging between 2.34 and 19.20 ng/L. The situation is comparable in Sweden, where imidacloprid was detected in 36 % of the points sampled by Kreuger et al. (2010). The Swedish guideline value of 13 ng/L was exceeded 21 times, with a maximum concentration of 15,000 ng/L, which is 1,154 times over the guideline value. Acetamiprid was also detected, exceeding the guideline value of 100 ng/L twice, with a maximum value of 410 ng/L. Concentration of imidacloprid at 1 μg/L was reported by Bacey (2003) in California groundwater. Concentration reaching 6.4 μg/L were measured from wells in potato-growing areas in Quebec with detection of imidacloprid and three of its metabolites in 35 % of these wells (Giroux 2003). Detections ranging from 0.2 to 7 μg/L were measured in New York State (US EPA 2008).

Fipronil was detected in the Mermentau and Calcasieu River Basins in the USA, in more than 78 % of water samples from the study area. The metabolites fipronil sulfone and fipronil sulfide were detected more often than the parent compound in 81.7 and 90.0 % of the samples, respectively (Mize et al. 2008). In an earlier report by Demcheck et al. (2004), the accumulation of fipronil degradates in sediment in the same area was reported (100 % of samples). Both authors report that higher concentrations of fipronil and its metabolites were connected to changes in aquatic invertebrate communities, notably a decrease in abundance and diversity. Contamination with fipronil has also an impact on fish as exemplified by Baird et al. (2013).

The contamination of groundwater is also a concern. With the large-scale use of these systemic insecticides and the increasing evidence of their presence in surface water, it should be taken into account that the time lapse between first application of a pesticide and its measured presence in groundwater is, on average, 20 years. Atrazine, for example, is only recently being discovered in groundwater despite having been registered in 1958. Detection of contamination of groundwater with neonicotinoids and fipronil is only a matter of time (Kurwadkar et al. 2013) as this is also the case for lindane (Gonçalves et al. 2007). This is supported by levels measured for thiamethoxam in 2008 and 2009 where several wells in Wisconsin had values above 1 μg/L, with a maximum at 9 μg/L (Huseth and Groves 2013, 2014). Following these results, imidacloprid (average, 0.79; range, 0.26–3.34 μg/L), clothianidin (average, 0.62; range, 0.21–3.34 μg/L), and thiamethoxam (average, 1.59; range, 0.20–8.93 μg/L) were detected at 23 monitoring locations over a 5-year period.

##### Exposure routes

Exposure of nontarget organisms in aqueous environments can take place through different scenarios. Baird et al. (2013) studied toxicity and exposure levels of fipronil on fathead minnow (*Pimephales promelas*), and stated that although waterborne fipronil can be toxic to larval fish, this would only be of concern at high concentrations. The authors conclude that it is the exposure through sediment that presents the real threat to aquatic organisms, including bioaccumulation of fipronil, fipronil sulfone, and/or fipronil sulfate in fish. The fact that systemic pesticides are more persistent in low-light conditions draws further attention to the importance of this exposure route.

Other exposure routes could include the use of contaminated water as drinking water. For example, honeybees (*Apis mellifera*) use water in the hive for cooling and for preparing liquid food for the brood (Kühnholz and Seeley 1997). In extreme conditions (desert), water foraging bees can collect water from up to 2 km from their colony (Visscher et al. 1996). EFSA (2012a) reports 20–42 L per colony per year, and up to 20 L a week or 2.9 L a day in summer. They draw attention to the lack of data on the exposure of honeybees to water through surface water, puddles, and in leaves and/or axils, and recommends that this should be taken into consideration when determining the level of exposure to honeybees.

#### Conclusion

The high to moderate solubility, leaching potential, and persistence of most of the neonicotinoids and fipronil pose a continuing and increasing risk to aqueous environments. Detections of (high) concentrations in groundwater and surface water are becoming more widespread around the globe. With an ever-increasing scale of use and a relatively high toxicity for aquatic invertebrates, severe impacts on aquatic ecosystems can be expected, and are indeed being discovered (Skrobialowski et al. 2004, cited by Mize et al. 2008; Goulson 2013; van Dijk et al. 2013; Pisa et al. 2014, this issue).

## Environmental fate and exposure in plants

### Introduction

The efficacy of neonicotinoid insecticides is due in part to the moderate to high water solubility (PPDB 2012); a factor which enhances the uptake and translocation of active ingredients. An advantage associated with using these systemic products is that treated plants are resistant to pests much longer than those treated with nonsystemic products (Dieckmann et al. 2010b).

Neonicotinoids and fipronil are taken up by plants, e.g., by the roots or the leaves, and then transported along the phloem or the xylem to distal tissues different from those where the product was applied (Nauen et al. 2001; Dieckmann et al. 2010a; Aajoud et al. 2008), including the flowers (Bonmatin et al. 2003, 2005b), their pollen (Bonmatin et al. 2007; Krupke et al. 2012), and nectar (Stoner and Eitzer 2012; Paradis et al. 2014). Thus, no matter where a pest or nontarget organism attacks the treated plant it is likely to come in contact with these chemicals. This chapter aims to provide an overview on the environmental fate of neonicotinoids and fipronil in plants and subsequent exposure routes for non-target organisms.

### Uptake by the roots and leaves

Prediction of translocation of pesticides in plants is difficult. Plant morphology and physiology as well as chemical properties of the specific compounds are highly variable and the mechanisms behind translocation processes are often poorly known (Trapp 2004). This chapter focuses on several physical-chemical characteristics of neonicotinoid insecticides and fipronil, aiming to describe the translocation of these pesticides within treated plants after their application.

Systemicity depends on the physical-chemical parameters of the chemicals including water solubility, the partition coefficient octanol/water (log *P*_ow_ or *K*_ow_) and the coefficient of dissociation (p*K*_a_). The values of these parameters for the molecules of interest (neonicotinoids and fipronil) can be found in Table 2. However, there are ways to render nonsystemic products, such as fipronil, systemic, by adding copolymers to the pesticide formulation (e.g., Dieckmann et al. 2010a, b; Ishaque et al. 2012).

**Table 2:** Physical-chemical parameters of neonicotinoids and fipronil determining their translocation capacity within the plant

Active substance	Molecular weight (g/mol)	Water solubility (g/L)	Octanol/water partition coefficient (log *P*_ow_)	Dissociation constant (p*K*_a_)
Fipronil	437.15	0.00378	3.75	No dissociation
Imidacloprid	255.7	0.61	0.57	No dissociation
Thiamethoxam	291.71	4.1	−0.13	No dissociation
Thiacloprid	252.72	0.184	1.26	No dissociation
Clothianidin	249.7	0.34	0.905	11.1
Acetamiprid	222.67	2.95	0.8	0.7
Nitempyram	270.72	590	−0.66	3.1
Dinotefuran	202.21	39.83	−0.549	12.6

#### Partition coefficient octanol/water (log *K*_ow_)

This parameter indicates the lipophilicity of substances which is related to the ability of substances to penetrate through bio-membranes (Trapp 2004). In order to enter into the plant, chemicals need to cross the plant cuticle. The coefficient cuticle/water is closely linked to the log *K*_ow_ (Trapp 2004). However, it is difficult to predict cuticle uptake as it depends on many other factors such as the chemical ingredient, the contact area, the cuticle surface, etc.

When used as root, soil, or seed applications, the sorption of organic chemicals to plant tissues depends on the root concentration factor (RCF) which is the ratio between the concentration in the root (g/g) and the concentration in solution (g/mL). The dependency of the RCF on the *K*_ow_ has been empirically estimated by Briggs et al. (1983). Maximal cuticle permeability occurs with neutral lipophilic compounds (Trapp 2004), log *K*_ow_ being around between 1 and 2.5. Compounds can be considered systemic when their partition coefficient octanol/water goes from 0.1 to 5.4 (Dieckmann et al. 2010a). Certain experts (ICPPR: International Commission for Plant-Pollinator Relationships, http://www.uoguelph.ca/icpbr/index.html) have proposed to consider a molecule as systemic if the partition coefficient lays underneath 4 because of hydrosolubility. A parameter that may influence the uptake of pesticides by the roots is the adsorption of chemicals by the soil. However, the final determination of the systemic character should be based on residue analyses or fate analyses in order to reduce uncertainties.

Similarly, when applied as foliar spray, the log *K*_ow_ and the concentration of the applied formulation also influence uptake via the leaves. Buchholz and Nauen (2002) describe two additional parameters that alter cuticle permeability of systemic insecticides: molecular mass and temperature. Molecules with high molecular mass at low temperatures tend to penetrate less (Baur et al. 1997). However, cuticle specific characteristics are determinant for pesticide uptake.

#### Dissociation coefficient (pK_a_)

This parameter indicates if the diluted form of the molecule is a weak or a strong acid. A p*K*_a_ < 4 indicates a strong acid, while p*K*_a_ > 5 indicates a weak one. It is important to note that the phloem pH of plants is around 8 and the xylem pH is around 5.5. Almost all systemic compounds are weak electrolytes (Trapp 2004). The p*K*_a_ of neonicotinoids and fipronil (many in their undissociated form) are shown in Table 2. Roots tend to show higher uptake rates at reduced pH (Rigitano et al. 1987), with uptake increasing around p*K*_a_ 3 and partition coefficients between 1 and 3.

Apart from the inherent systemic properties exhibited by pesticide active substances, a wide variety of options have been patented in order to increase uptake—by increasing systemicity, solubility, etc.—which are mainly based on a co-formulation of pesticides with copolymers (e.g., Dieckmann et al. 2010a, b; Ishaque et al. 2012). Cell wall permeability of pesticides might also be increased due to the use of polymers (Chamberlain 1992). As a result, uptake by plants, either via the roots or the leaves, is enhanced when polymers are applied.

Imidacloprid and acetamiprid show different uptake capacities by cabbage (70–80 % recovered activity at day 1) and cotton (30–40 % penetration at day 1), respectively. However, both compounds still exhibit 100 % efficacy 12 days following foliar application (Buchholz and Nauen 2002). Non-absorbed active ingredients remain on the surface of the leaves or get associated with epicuticular waxes. Eventually, given their water solubility, these residues could be redissolved into guttation water or morning dew water and could be available to insects.

Imidacloprid uptake via the roots has been shown to range from 1.6 to 20 %, for aubergine and corn, respectively (Sur and Stork 2003). The remainder of the applied active substances is left behind in the soil and should be explored to determine its environmental fate.

The draft assessment report (DAR) of thiamethoxam in 2001 (EFSA 2013b) includes studies of distribution and metabolism of ^14^C-oxadiazin- and ^14^C-thiazol-thiamethoxam investigated in corn (seed treatment); pear and cucumber (foliar application); lettuce, potato, tobacco, and rice (soil and foliar treatment). All applications show high and fast uptake (e.g., 23 % recovered activity in the plant within day 1, 27 % of the applied amount being found after 28 h in leaves), where the product is continuously taken up from the soil reservoir for at least 100 days. The metabolism of thiamethoxam is very rapid, both inside the plant and following foliar application (photodegradation, 30 % degradation in 12 h of sun). Clothianidin is the main metabolite of this active ingredient.

Field experiments show that neonicotinoids tend to have good systemic properties (Maienfisch et al. 2001; Sur and Stork 2003). Fipronil is often described as being less systemic than the neonicotinoids. However, uptake and translocation of this active ingredient following granular application on sugar beets has been confirmed (fipronil DAR from EFSA 2013d). Following a rate application of 2,000 g a.i./ha, 10 times more recovered activity was found in leaves (0.66 mg/kg fipronil equivalents) than in roots 6 months after soil treatment, where 0.06 mg/kg fipronil equivalents were found. In the roots, fipronil sulphone was the main component (64 % of total radioactive residue (TRR), followed by fipronil (14 % TRR) and its amide derivative (RPA200766) (5 % TRR)), while the leaves contained fipronil sulphone (31 % TRR), followed by RPA105320 (18 % TRR) and to a lesser extent MB45950, MB45897, and the amide derivative (less than 0.03 μg/g and 4 % TRR) (see Simon-Delso et al. 2014 for definition of metabolites). Fipronil was found at lower amounts in these leaves. Experiments carried out on corn (420 g a.i./ha) have also shown the systemic activity of fipronil with 0.16, 0.18 and 3.93 ppm of fipronil equivalents being recovered 42, 98, and 106 days after treatment, respectively. Fipronil, its sulfone derivative and its amide derivative were the main components found (fipronil DAR from EFSA 2013d).

### Transport of products within the plant

When systemic products are taken up by the roots, the acropetal translocation of pesticides via the xylem sap follows. Translocation into the shoots is described by the transpiration stream concentration factor (TSCF), which is the ratio between the concentration in xylem sap (g/mL) and the concentration in the solution (g/mL). Briggs et al. (1983) found that the translocation of neutral chemicals is most effective for compounds with intermediate lipophilicity. Pesticides with intermediate lipophilicity tend to be xylem mobile. For this reason, they tend to accumulate in the stem cells and show a decreasing acropetal gradient. However, if polarity or lipophilicity increases, permeability tends to decrease (Briggs et al. 1983). Woody stems retain chemicals more effectively than younger stems due to the lignin content of cells.

The p*K*_a_ of imidacloprid (14) indicates that it remains in its undissociated form, despite any pH variations within the plant, diffusing freely within the plant transportation system. As a result, a good membrane penetration and a high xylem mobility can be predicted for imidacloprid (log *K*_ow_ = 0.57). Imidacloprid is therefore expected to be found in the xylem and not in the phloem because of the weak acidity/nondissociation and a TSCF of 0.6 (Sur and Stork 2003). Translocation into the xylem is mainly driven by water flow from the roots to the upper parts of the plant. However, its polarity and solubility in water (0.61 g/L) results in limited retention by tissues and no accumulation in roots (Alsayeda et al. 2008). Thiamethoxam is also likely to be translocated (mainly acropetally) via the xylem sap (Maienfisch et al. 2001).

Theoretically, systemic products taken up by the leaves circulate to the rest of the plants mainly via phloem transport. However, translaminar and acropetal mobility have also been observed, with radiolabeled imidacloprid being shown to move toward the leaf tips and margins following foliar application (data from DAR). Aphid mortality tests confirmed the rapid systemic translocation of imidacloprid and acetamiprid within 1 day of application. Following foliar application, thiamethoxam also tends to accumulate in the leaf tips. This might be the reason that guttation water (excreted from the leaf margin) is so concentrated with neonicotinoid active ingredients (Girolami et al. 2009).

Phloem mobility tends to occur with compounds of intermediate lipophilicity (log *K*_ow_ between 1 and 3) and weak acidity (p*K*_a_ between 3 and 6) (Rigitano et al. 1987; Trapp 2004). The ion trap theory has been proposed for polar undissociated molecules, which exhibit intermediate permeability through cell walls and being translocated in the phloem immediately after application.

Imidacloprid exhibits xylem translocation, meaning that it is found mainly in the shoots and leaves. Following foliar application of a spray formulation of imidacloprid, a maximum of 0.1 % recovered activity could be found in fruits (Sur and Stork 2003). Imidacloprid is not translocated via the phloem; therefore, in theory, the amount of residues found in roots, fruits, and storage organs should be minimal (imidacloprid DAR 2006). However, some of its metabolites meet the physical-chemical conditions to be basipetally translocated, as for example 6-chloronicotinic acid. As a result, this compound or others with the same characteristics can be found in plant parts different from the site of application (Chamberlain et al. 1995).

Soil applications to potato and cucumber confirm the systemic property and acropetal mobility of thiamethoxam and show that the degree of uptake depends upon the method of application as well as the plant species and that this product tends to accumulate at the leaf tips and borders (thiamethoxam DAR). Leaf application confirms the acropetal translocation with relatively high concentrations of thiamethoxam in leaf tips. Small basipetal mobility can also be observed confirming phloem mobility of this compound.

In fact, the amount of imidacloprid, thiamethoxam, clothianidin, or their active metabolites translocated by the phloem seems to be high enough to achieve effective aphid mortality, considering that these insects are mainly phloem feeders (Nauen et al. 2003).

### Exposure

As shown in Simon-Delso et al. (2014, this issue), the systemic properties of neonicotinoids and fipronil ensure that these compounds are taken up in all parts of the treated plant. There is much variability in pesticide dissipation (half-lives) in plants, as shown in a review by Fantke and Juraske (2013). The authors examined 811 scientific literature sources providing 4,513 dissipation times (half-lives) of 346 pesticides, measured in 183 plant species.

#### Foliage

Exposure of nontarget organisms to neonicotinoids and fipronil can occur via the ingestion of unintentionally treated plant parts (i.e., leaves, flowers, etc.). Depending on the application method, potential exposure by consuming contaminated foliage can take place after seed sowing or after spray treatment and exposure could potentially persist up to point of harvest or beyond. This risk of exposure will differ with crop type and chemical application method. In agricultural production, aerial part of crops is often a major by-product or waste component following the harvest of various crops. These products are often sold and used for varying purposes (livestock feed, industrial products, biofuel production, etc.) but may also be left in or next to the field where the crop is harvested. Again, depending on the crop and application method, this may be an exposure route for nontarget organisms. For example, Bonmatin et al. (2005b) evaluated imidacloprid content in the stems and leaves of maize treated with imidacloprid (Gaucho seed treatment, 1 mg/seed). The average concentration detected in the mixture of stems and leaves at the time of tasseling was 4.1 μg/kg, with 76 % of the samples containing more than 1 μg/kg.

Another example is sugar beet foliage, which is separated from the beet during harvesting and may be left on the field. Westwood et al. (1998) found that 3 weeks after spray treatment at a rate of 0.9 mg/seed of imidacloprid, leaves of sugar beet seedlings contained an average of 15.2 μg/kg. Rouchaud et al. (1994) applied imidacloprid in the form of a seed dressing at 90 g/ha. The highest concentration of 12.4 mg/kg fresh weight was found in sugar beet leaves in the first week after sowing and concentrations remained greater than 1 mg/kg for 80 days after sowing. However, imidacloprid was not detected in the roots or leaves of sugar beets at harvest (LOD, 10 μg/kg). Similarly, imidacloprid was not detected in grape leaves at the time of harvest (Mohapatra et al. 2010).

These varying results indicate that exposure of nontarget organisms to parent compounds via contact with treated foliage will depend on the crop, application method, and also the time period following treatment. However, the levels of metabolites are often not taken into account. Sur and Stork (2003) found the main metabolites of imidacloprid in a wide variety of crops including maize, eggplant, cotton, potatoes, and rice. These included the olefin and hydroxyl metabolites of imidacloprid, which are known to have similar levels of toxicity in *A. mellifera* as the parent compound (Suchail et al. 2001). Based on the overview of parent compounds and metabolites found in nectar and pollen (*vide supra*), contact with or ingestion of treated foliage may indeed represent a route of exposure to nontarget organisms. This is further substantiated in the case of fipronil-contaminated silage (maize, dry material) which was found to contain 0.30 ng/g of fipronil and 0.13 ng/g of the metabolite sulfone-fipronil (sulfide-fipronil < 0.025 ng/g). Furthermore, this indirectly led to the contamination of cow milk with sulfone-fipronil, at an average value of 0.14 ± 0.05 μg/L (0.14 ± 0.05 ppt) (Le Faouder et al. 2007).

#### Tree treatment

Imidacloprid is currently used to protect trees against wood-boring insects such as the emerald ash borer (*Agrilus planipennis fairmare*) or the Asian longhorned beetle (*Anoplophora glabripennis motschulsky*). It can be applied either through soil injection (drenching) at the base of the tree or through trunk injection, with the systemic action of imidacloprid providing protection for the entire tree (Cowles et al. 2006; Poland et al. 2006; Kreutzweiser et al. 2009).

Cowles et al. (2006) studied the concentrations of imidacloprid in Hemlock (*Tsuga* spp.) needles, twigs, and sap using soil and trunk injection methods and found residues after 1 month and up to 3 years after application. The detected concentration of imidacloprid in needles and twigs ranged from stable to increasing at times during the 3 years after application. This was more often the case when a soil injection was used, possibly due to continued uptake through the roots. These findings indicate the relative stability of imidacloprid once it is absorbed by the tree. Tattar et al. (1998) studied imidacloprid translocation in Eastern Hemlock (*Tsuga canadensis*), White Pine (*Pinus strobus*), and Pin Oak (*Quercus palustris*) using soil and trunk applications. Although a continuous increase in imidacloprid concentration was observed in *Q. palustris* and *T. canadensis* after soil application, the restricted sample size (*n =* 6) and sampling period render these results inconclusive with regard to the persistence of imidacloprid in these tree species. In addition, the concentration of imidacloprid in *P. strobus* needles began to decrease 12 weeks after treatment, indicating that the degradation of imidacloprid in tree foliage may be species-dependent. Multiple factors can be hypothesized to play a role in this mechanism including exposure to light, temperature differences, and the efficiency of translocation within the tree.

The efficacy of fipronil, acetamiprid, and imidacloprid as tree treatments were studied by Grosman and Upton (2006). In contrast to imidacloprid, fipronil appeared to take more than 1 month to disperse throughout all tree parts in *Pinus taeda* L. The authors hypothesized that fipronil could protect these trees for more than 1 year, again indicating this compound may be quite stable once acquired by tree tissues. The use of other neonicotinoids for tree treatment has not been documented, and therefore cannot be taken into account.

#### Guttation and related risk for honeybees

Guttation (Burgerstein 1887) is a natural phenomenon observed in a wide range of plant species (Bugbee and Koerner 2002; Singh and Singh 2013). Guttations are water droplets that are exuded from specific secretory tissues (hydathodes) located along the margins and tips of leaves in response to root pressure or excess water conditions (Goatley and Lewis 1966; Koulman et al. 2007; Katsuhara et al. 2008; Duby and Boutry 2009). These aqueous solutions may contain a variety of both organic and inorganic compounds (Singh et al. 2009a; Singh et al. 2009b). This phenomenon is mainly observed during the first hours of the morning; however, it can also occur throughout the day depending on environmental conditions. Guttations are also a mechanism by which plants regulate leaf turgidity (Curtis 1944; Knipfer et al. 2011).

In a comprehensive review of guttations, Singh and Singh (2013) reported that different secretory organs such as nectaries, hydathodes, and trichomes, produce secretions with varying functions including the disposal of solutes, improvement of hormone and nutrient acquisition, attraction (i.e., for pollination) or repulsion (for defense purposes). However, these liquid secretions are not to be confused with guttations, which are much more prominent. In addition, adult plants do not produce guttations regularly, while young plants tend to produce guttations frequently and at greater volumes.

As for the presence of insecticide residues in guttations, adult plants are normally treated with spray formulations which lead to active ingredient concentrations in the ppb range or below (Shawki et al. 2005). Conversely, guttations produced by seedlings grown from coated seeds can reach insecticide concentrations of hundreds of ppm (Girolami et al. 2009; Tapparo et al. 2011). In our opinion, it is crucial to distinguish the risk posed by contaminated guttations arising from young versus mature plants, so as to accurately estimate the risk of acute intoxication for bees via ingestion and/or contact with guttations from insecticide-treated plants such as cereals. Moreover, in regions dominated by cereal production, the land area devoted to these crops is often greater than that of other noncereal crops. As a consequence, cereal guttations (i.e., maize guttations) may be produced across millions of hectares (Girolami et al. 2009).

The production of guttations by corn plants in southern Europe occurs during the first 3 weeks after seedling emergence. The produced amount is not well quantified; a first estimation indicates that each seedling produces 0.1–0.3 mL per day of guttations during the initial period of high guttation production, and less than 0.1 mL per day during the final days in which the phenomenon occurs (Girolami et al. 2009).

These aqueous solutions have not been considered as a potential source of contamination for insects since 2005. Shawki et al. (2005) evaluated the guttations of adult plants sprayed with an organophosphate insecticide and detected sub-ppb levels of active ingredient in droplets. The translocation of neonicotinoid insecticides from coated seeds to young plant guttations (at ppm levels) was observed for the first time in maize seedlings in spring 2008 (Girolami et al. 2009). Because neonicotinoids are water soluble and circulate systemically, residues or high concentrations of active ingredients can be found in guttation drops (Tapparo et al. 2011). The time at which samples are collected for analysis can strongly influence the detection of neonicotinoids in guttations. For example, the same authors show that 1 month after sowing, the concentration of insecticides in guttations decreases dramatically to a few ppb.

In general, neonicotinoid concentrations in guttation drops of corn seedlings show very high variability, and are only partially influenced by the amount of insecticide coating on the surface of the seed (Tapparo et al. 2011). The systemic properties and chemical stability of neonicotinoids in the soil and also within the plant seem to have strong effects on concentrations in guttation droplets. Values of a few ppm have been measured in Northern Europe (Reetz et al. 2011; Pistorius et al. 2012) while values of 10–1,000 ppm have been observed for at least 2 weeks by Girolami and co-workers in Italy (Girolami et al. 2009; Tapparo et al. 2011).

In addition, several climatic variables can affect neonicotinoid concentration in guttation drops of corn seedlings. Preliminary experiments in Italy demonstrate that under high humidity conditions (close to saturation, a situation that often occurs during the morning in spring) insecticide concentrations can be 10 times lower than those observed in guttations formed during the following sunny hours. This difference could be relevant especially in the warmer area of Europe. Moreover, guttation production by corn seedlings may be dramatically reduced or ended under low humidity conditions (RH 50–60 %). Rain can reduce the concentration of insecticide in guttations by about 10 times with respect to the values observed the day before a rainfall event. Sunny conditions and a moderate wind can promote water evaporation and affect the concentration of insecticide in guttation drops. On the contrary, strong winds can dislodge droplets off leaves, eliminating any concentration effects that would otherwise occur if droplets remained on the leaves. Finally, soil moisture and composition only moderately affect the insecticide concentration of guttation droplets (APENET 2011), suggesting that air humidity is a significant environmental factor to consider in the case of guttations.

Guttations contaminated by high levels of neonicotinoids can also be produced by other insecticides. For instance, clothianidin can be applied in granular form directly to the soil during corn sowing, giving concentration levels of the same order of magnitude (or slightly lower) of those observed in guttations produced from coated seeds (Pistorius et al. 2012) and with almost identical levels of acute toxicity for bees. Another interesting case concerns the massive use of insecticide applied directly to the soil with irrigation water (fertigation) and inducing concentrations of neonicotinoids in guttations of cucurbitaceae in the range of a few ppm (Stoner and Eitzer 2012; Hoffman and Castle 2012). Thus, environmental contamination is possible, but it is not comparable to guttations from young plants obtained from coated seeds.

It is worth noting that corn guttations may show concentrations of insecticide higher than 1,000 ppm (mg/L); these values match the insecticide content (about 1 ‰) of the aqueous solutions used for foliar spray treatments. Despite the high levels of contamination, the influence of toxic guttations on spring losses of bees appears to be limited, as reported in Girolami et al. (2009) and Tapparo et al. (2011). Generally, bees collect water from spontaneous vegetation, well before maize emergence, and they do not require guttation droplets from maize fields. Although some individual explorer may drink guttations from the maize field, it would die in a few minutes (due to high pesticide concentration, lethal for bees even by contact only) and not have the time to communicate the presence of the water source to the colony. This does not exclude that the large extensions of poisonous drops cannot constitute a problem for other pollinators that nest in the ground (*Andrena* spp., *Halictus* spp.) or have an erratic behavior (*Bombus* spp. for example), resulting from the fact that they do not have communication ability through dance like bees. Those species would be killed by contact with contaminated guttations.

Concerning other systemic insecticides, the absence of relevant literature hinders any solid conclusion. As preliminary data, we can report that guttations of corn seedlings obtained from seeds coated with fipronil contain lower concentrations of the insecticide (ppb levels) with respect to those obtained with neonicotinoid seed coating. Nevertheless, if administered to bees (solution with 15 % honey), these guttations are lethal within minutes, indicating the possible presence of metabolites with high acute toxicity (Girolami et al. 2009).

#### Resin (propolis)

Resin is harvested by honeybees (*A. mellifera*) and used as propolis for sealing holes and evening out surfaces within the beehive. Sources of propolis are tree buds and exudates from plants. Although pesticide residues have been reported in propolis, no information is available about neonicotinoids or fipronil.

Pareja et al. (2011) hypothesize that sunflower resin can be used by honeybees, thereby making it a possible source of pesticide exposure. The authors took five propolis samples from depopulated hives located near sunflower crops, which were also the only crops in the area to be previously treated with imidacloprid. Imidacloprid was detected in two of the samples at 20 and 100 ng/g, respectively, which supports the hypothesis that sunflower resin may be a potential exposure route for honeybees and other nontarget organisms that collect resin.

#### Presence in plant reproductive organs and fruits

Intake of systemic insecticides through residues in fruits and vegetables is a potential risk to invertebrates and vertebrates alike. Fruit and vegetables deemed unfit for human consumption may be discarded in piles that are easily accessible to various organisms. In addition, inadequate storage methods may provide further means of exposure to these insecticides.

The concentration of residues in the reproductive organs of plants following treatment varies with plant species and application method. Translocation studies show imidacloprid residues in plant reproductive organs ranging from 0.7 to 12 % of the originally applied soil treatments in rice and potato plants, respectively (Sur and Stork 2003). Sunflower treated with fipronil through soil treatment shows 0.2 % of the applied product in flower heads and seeds (EFSA 2013d, fipronil DAR).

Concerns regarding the contamination of fruits and vegetables with regard to human health are beyond the scope of the present study. However, the translocation of residues of systemic products into fruits can be achieved either by their transport through the xylem or phloem (Alsayeda et al. 2008), although the mechanisms of accumulation in fruits are not yet fully understood. Juraske et al. (2009) studied the human intake fraction of imidacloprid for unwashed tomatoes and found that it varies between 10^−2^ and 10^−3^ (kg_ingested_/kg_applied_) depending on the time of consumption. This was the case for tomato plants treated with the recommended doses in spray application as well as chemigation. Sur and Stork (2003) found that tomato and apple exhibit 21 and 28 % recovery of applied compounds following a foliar application. More than two thirds of this recovery was located on the surface of the fruits. A study by Zywitz et al. (2004), examined a range of fruit and vegetable groups for which neonicotinoid residues could be detected (LOD = 3 ng/g) and quantified (limits of quantification (LOQ) = 5 ng/g) (Table 3). Fruiting vegetables (tomatoes, pepper, cucumbers, courgettes, and melon) exhibited the highest number of positive samples (46.7 %), followed by leafy vegetables and fresh herbs (lettuce, cress, spinach, dill, chives, and parsley; 10 %), stone fruits (peach, nectarine, apricot, and cherry; 4.5 %), pome fruits (apple and pear; 2.9 %), and berries (strawberry, raspberry, currant, blueberry, and grape; 2.2 %). No information was provided on the method of application of neonicotinoids or the doses used. More recently, 22 % of fruits sampled in India showed the presence of imidacloprid and 2 % were above the maximum residue level (MRL) (Kapoor et al. 2013). A similar situation has been described in Turkey, with levels of acetamiprid in vegetables occurring above the allowable MRL (Sungur and Tunur 2012).

**Table 3:** Quantity of positive samples of neonicotinoids in multiple fruit groups

Group	Commodities analyzed	Nb. of samples	Nb. positive samples	Nb. samples > MRL
Citrus fruits	Lemon, orange, mandarin, grape fruit	177	2	0
Stone fruits	Peach, nectarine, apricot, cherry	111	5 (4.5 %)	0
Pome fruits	Apple, pear	175	5 (2.9 %)	0
Berries	Strawberry, raspberry, currant, blueberry, grape	556	12 (2.2 %)	3 (0.5 %)
Tropical and subtropical fruits	Pineapple, kiwi, kaki, mango, kumquat	101	1	1
Leafy vegetables and fresh herbs	Lettuce, cress, spinach, dill, chives, parsley	231	24 (10.4 %)	3 (1.3 %)
Fruiting vegetables	Tomato, pepper, aubergine, courgette, melon, cucumber, chili pepper	540	252 (46.7 %)	104 (19.3 %)
Brassica vegetables	Cauliflower, Chinese cabbage, Brussels sprout, kohlrabi, white cabbage	47	1	0
Root and tuber vegetables	Carrot, radish, swede	39	0	0
Dietary foods, cereals and cereal products	Maize, wheat, commeal, maize semolina, bran, rice and other	50	0	0
Legume and stem vegetables	Asparagus, bean, pea, celery	33	0	0
Miscellaneous	Rape, tea, dried fruit, leek, must mash, potato, (concentrated) fruit juice and other	64	0	0

Source, Zywitz et al. (2004)

The contamination of nectar and pollen following treatment with neonicotinoids and fipronil is well known. Sunflowers seed-treated with imidacloprid have been shown to contain an average of 4.6 ng/g in the stems and leaves, 8 ng/g in flowers, and 3 ng/g in pollen (Bonmatin et al. 2003). In maize, Bonmatin et al. (2005b), showed a mean recovery of 4.1 ng/g in stems and leaves (max 10 ppb), 6.6 ng/g in male flowers (panicles, max 33.6 ng/g), and 2.1 ng/g in pollen (max 18 ng/g) following seed dressing at a rate of 1 mg/seed. Monitoring studies in Austria reported thiacloprid levels in nectar or honey to be between 11.1 and 81.2 ng/g (Tanner 2010). An extensive review of the contamination of pollen and nectar is given below.

#### Pollen and nectar

Pollen and nectar from flowers are collected by bees and form an integral component of their diet. Pollen and nectar also constitute the feeding resources of many nontarget insects of less economic importance. The contamination of pollen and nectar has been measured mainly for honeybees and bumble bees. However, these measurements also represent valuable starting points for assessing exposure risks of other nontarget species.

Pollen can be sampled in different forms—it can be obtained directly from flowers, by trapping from bee hives (bee-collected pollen pellets), or from bee bread (bee-mixed pollen and nectar). Nectar is converted by bees into raw/fresh honey and it is also a component of bee bread. Obviously, contamination of these matrices depends heavily on the presence of residues in flowers (Bonmatin et al. 2003; Aajoud et al. 2008) but also upon the presence of residues found and collected directly in the environment of the bees (water, dust, etc.). Residues are defined as active ingredients used in crops and/or their active metabolites (Simon-Delso et al. 2014, this issue), although other compounds may be present (adjuvants or synergistic compounds). These other compounds are generally not considered for analysis or assessment, but could be of importance for toxicity toward nontarget species (Mesnage et al. 2014). However, it is often only the active ingredient which is measured in the majority of cases. Residues contained in pollen and nectar can be transformed or metabolized by bees, inside and outside the hive. Such complex processes are not well understood. Furthermore, these residues can cross-contaminate other matrices (bees, pollen, bee bread, nectar, honey, wax, propolis, royal jelly, etc.) (Rortais et al. 2005; Chauzat et al. 2006; Mullin et al. 2010). The routes of exposure for honeybees, bumble bees, and solitary bees were identified by the European Food Safety Authority (EFSA 2012a) and ranked from 0 (no route of exposure) to 4 (highly relevant route of exposure). Although some of these routes will need to be re-evaluated as new evidence comes to light, nectar and honey, pollen, and bee bread all share the highest scores and are therefore the most likely routes of exposure for bees.

##### Assessment

The ecological risks of active ingredients are assessed using the hazard quotient (HQ) calculation. This approach estimates whether harmful effects of the contaminate in question may occur in the environment by comparing the Predicted Environmental Concentrations (PEC) to the Predicted No Effect Concentration (PNEC). HQ calculations do not consider the mode of insecticide application, the systemic properties, routes of exposure, or the persistence or metabolism of pesticides. Historically, these calculations have been inaccurate due to a lack of adequate analytical techniques for the quantification of residues in matrices like pollen and/or nectar. This was the case for imidacloprid and fipronil in the 1990s—the initial risk assessment assumed that flowers were not significantly contaminated with respect to the LD_50_ values for bees and so the PEC was underestimated at the time of registration (Maxim and van der Sluijs 2007). However, with the improvement of analytical techniques, the detection of residues in pollen/beebread and nectar/honey have become more accurate (Bonmatin et al. 2005a; Dively and Kamel 2012; Paradis et al. 2014), and show that the PEC values are actually significantly higher. Meanwhile, new understanding of the sublethal and chronic exposure effects on bees has improved the PNEC value, and demonstrates that this value was clearly overestimated during the registration of these products (Suchail et al. 2001; Whitehorn et al. 2012). It was only in the early 2000s that assessments were conducted for imidacloprid using accurate data (Rortais et al. 2005; Halm et al. 2006). This work considered both (1) different exposure pathways and (2) relative needs in food among various castes of honeybees (foragers, nurses, larvae, winter bees, etc.).

The risk assessment of pesticides on bees has recently been completed in the EU. Currently, the risk of pesticides to bumble bees and solitary bees is taken into account (EFSA 2012a; EFSA 2013f) and different exposure forms are considered: (a) ingestion, (b) contact, and (c) inhalation. Additionally, bees are now assessed for (1) exposure inside the hive including food (mainly honey and bee bread), nest (including wax and propolis), and other bee products and (2) exposure outside the hive including water, plants (considering several matrices such as nectar and pollen as a food supply), guttation, air, dust, soil, etc. The same approach could be used for any other species feeding on pollen and/or nectar.

##### Variability

One of the main difficulties is the variability of measured data in these relevant matrices which depends significantly on the dose and mode of treatment, the studied crop, season, location, soil, weather, time, bees, etc. Even different crop varieties can induce significant variability in the residue content of pollen and nectar (Bonmatin et al. 2007). Additional sources of variability include variations in the amount of contaminated versus uncontaminated food harvested by bees (e.g., the proportion of treated pollen/total pollen and the proportion of treated nectar/total nectar); differences in metabolism between foragers and in-hive bees; the availability of alternative plant resources; the “filter” effects made by bees (e.g., trapped pollen is only brought back by nonlost foragers); the distance between treated crops and hives; effects of mixture (e.g., mixing nectar and pollen to produce bee bread) and the effects of concentration (e.g., reducing water content to produce honey from nectar); this list being non-exhaustive. Furthermore, measurements are not always performed on the same matrices or are influenced by the choice of samples and their location (experimental area) by the experimenters, which make comparisons of risk difficult. This is particularly relevant for water contamination, as water resources can differ significantly in their composition (surface water, ephemeral pooling, guttation etc.; EFSA 2013f) and because the concentration of contaminates in surface water can vary within the same area of foraging, from a few nanogram per liter (ppt) to a few nanogram per milliliter (ppb) (Starner and Goh 2012; Van Dijk et al. 2013; Goulson 2013; Main et al. 2014; Bonmatin, personal communication).

The contamination of fresh and stored honey originates from the presence of residues in nectar. Honey in beehives can be less contaminated than nectar. This situation was reported from sunflowers treated by seed dressing (Schmuck et al. 2001), but could have been due to a dilution effect, whereby mixture of treated and untreated nectar yields lower levels of contamination, as in the case of mixing pollen (*vide supra*). The opposite situation has also been described for citrus trees treated with soil applications (Byrne et al. 2014). Although the sum of processes remains poorly understood, it is known that there is an initial metabolism during transport and diverse chemical reactions and processing are conducted by workers—where the concentration factor is affected by the amount of water in the nectar (Winterlin et al. 1973) and by degradation over time leading to metabolites (Simon-Delso et al. 2014, this issue). Because foragers and in-hive bees participate in these metabolic processes, it can be assumed that in cases of high contamination of nectar, honey would not be stored in the hive so efficiently, due to deleterious effects on the global functioning of the beehive (Bogdanov 2006; EFSA 2012a).

In pollen, differences have been reported between samples directly taken from crops and pollen pellets brought back by bees to the beehive. These differences in contamination are mainly due to significant dilution effects when bees mix pollen from treated crops with that of untreated crops (Bonmatin et al. 2003, 2005b). Furthermore, when pollen is stored in the beehive to constitute bee bread, a range of chemical and biochemical processes occur which can contribute to the differences in residue levels between pollen types.

Another important source of variability comes directly from sampling protocols and analytical methods. It is clear that the latter are not harmonized, as evidenced earlier by the calculation of the HQ values. In the early 1990s, analytical techniques had not been improved sufficiently to measure contamination levels in the range of nanograms per gram (ppb). LOD and LOQ were higher than at the present time, by 2 orders of magnitude. Chromatography was generally coupled to a less sensitive detection system than those used currently (e.g., UV/Vis spectroscopy versus mass-tandem spectrometry) and the ambiguous statement “nd” (not detected) often suggested the absence of residues. Additionally, it was usually the stems and leaves which were analyzed, flowers being analyzed to a lesser extent. Nectar and pollen were rarely analyzed because extraction methods and detection methods were not efficient or sensitive enough for these particular matrices. More sensitive methods should have been set up more quickly by stakeholders.

The use of improved extraction methods and high-performance chromatography coupled with tandem-mass spectrometry allowed LOQ values to reach the range of 1 ng/g in the early 2000s. These methods were fully validated for the matrices of interest, with an LOD of a few tenths of ppb (Schmuck et al. 2001; Laurent and Rathahao 2003; Bonmatin et al. 2003; Chauzat et al. 2006; Mullin et al. 2010; Wiest et al. 2011; Paradis et al. 2014). Analysis can be further refined by focusing on one compound or a very limited number of compounds within a chemical class. This results in a significantly lower LOD and LOQ than normal screening methods, which are designed for numerous active ingredients. Moreover, extraction yields can be relatively low for some compounds in screening methods, and results are often underestimated because published data are generally not corrected with respect to the yield for each compound. Also, general screening methods are not relevant for risk assessment because this strategy aims to identify and quantify as many active ingredients as possible regardless of whether the active ingredients are pertinent to agricultural practices or not. For these reasons, risk assessment should always use specific targeted methods, whereas screening methods are more appropriate for gaining initial evidence of contamination (e.g., in unspecific monitoring studies). Recently, intermediate multiresidue methods (analyzing about 10 to 100 active materials) were published and present the advantage of being sensitive over a relatively wide range of residues in matrices such as nectar or honey (Wiest et al. 2011; Paradis et al. 2014). These methods are far better designed for detecting multiple exposures of bees than for risk assessment of one pesticide and are very useful in determining the presence of several pesticides within the same class of chemicals (e.g., neonicotinoids) or between various chemical classes (nicotinoids, phenylpyrazoles, and pyrethroids for instance). This is of particular interest when considering the possibility of additive toxicity or, in some cases, potential synergies.

For all the reasons listed above, it is not surprising that such high variability exists in the measurement of residues in the relevant matrices and this justifies the need for assessments to be based on the worst case scenario when data are lacking. However, there now exists for pollen/beebread and nectar/honey a body of data which allows for defining ranges of contamination of these matrices by the neonicotinoids and fipronil. Because this description is not limited to honeybees, this review focuses on the common food supply that can induce oral and contact toxicity to various types of pollinators.

##### Pollen and bee bread

Data reported by recent scientific reviews, scientific literature, some relevant Draft Assessment Reports (DAR) and other relevant reports, are presented in Table 4 (Johnson et al. 2010; EFSA 2012a; Thompson 2012; EFSA 2013a, c, e; Sanchez-Bayo and Goka 2014). These recent reviews were undertaken to assess pesticide residue levels including neonicotinoids and fipronil. To avoid repetition in the data (e.g., data issuing from citations in cascade), we indicate the original sources in Tables 4 and 5.

**Table 4:** Residues (neonicotinoids and fipronil) in pollen or in pollen-derived matrices (pollen/beebread)

Insecticide^a^	Detection rate^b^ (%)	Range^c^ (ng/g)	Mean^d^ or magnitude^e,f^ (ng/g)	Maximum^f^ (ng/g)	Reference^g^
Acetamiprid	24.1	1–1,000	3	134	Sanchez-Bayo and Goka (2014)
	45	0.1–100	4.1	26.1	Pohorecka et al. (2012)
	3.1	10–1,000	59.3	134	Mullin et al. (2010)
Clothianidin	11	1–100	9.4	41.2	Sanchez-Bayo and Goka (2014)
		0.1–100	0.1^h^ to 17.1^h^	21.1^h^	Dively and Kamel (2012)
		1–10	1^i^ to 4^i^	7	Pilling et al. (2014)
	11	1–10	1.8	3.7	Pohorecka et al. (2012)
		1–100	3.9	10.7	Krupke et al. (2012)
		1–100			In EFSA (2013a):
			7.38-	36.88	See estimate for maize
			5.95-	19.04	See estimate for rape
				3.29	See estimate for sunflower
				15	See Schöning 2005 (DAR)
		1–10		2.59	Cutler and Scott-Dupree (2007)
		1–10		2.8	Scott-Dupree and Spivak (2001)
		1–10			In EFSA (2012a):
				10.4	See Nikolakis et al. (2009) (DAR)
			2.6-	2.9	See Maus and Schöening (2001) (DAR)
				4.1	See Schmuck and Schöening (2001a) (DAR)
				3.3	See Schmuck and Schöening (2000b) (DAR)
				2.5	See Maus and Schöening (2001c) (DAR)
				3.1	See Schmuck and Schöening (2001d) (DAR)
				5.4	See Maus and Schöening (2001e) (DAR)
			3.3-	6.2	See Maus and Schöening (2001f, g) (DAR)
Dinotefuran	1	10–1,000	45.3	168.1	Sanchez-Bayo and Goka (2014)
	100	10–1,000	11.2 to 88.3 + 17.1^j^	147 + 21.1^j^	Dively and Kamel (2012)
	1	1–10	4	7.6	Stoner and Eitzer (2013)
Imidacloprid	16.2	1–1,000	19.7	912	Sanchez-Bayo and Goka (2014)
		0.1–1,000	0.1 to 80.2 + 19.1^k^	101 + 27.5^k^	Dively and Kamel (2012)
	9.1	1–1,000	30.8	216	Rennich et al. (2012)
	2.9	1–1,000	39	206 + 554^l^ + 152^l^	Mullin et al. (2010)
	40.5	0.1–10	0.9	5.7	Chauzat et al. (2011)
		1–100	14	28	Stoner and Eitzer (2012)
	12.1	1–100	5.2 + 5.6^l^	70 + 5.6^l^	Stoner and Eitzer (2013)
		10–100	13	36	Laurent and Rathahao (2003)
	87.2	0.1–100	2.1	18	Bonmatin et al. (2005)
		1–100	9.39	10.2	Byrne et al. (2014)
		1–100	2.6	12	Wiest et al. (2011)
	83	0.1–100	3	11	Bonmatin et al. (2003)
		1–100			In EFSA (2013c):
			3-	15	See Stork (1999) (Germany 2005, DAR)
			3.45-	4.6	See Germany 2005 (DAR)
		1–10			In EFSA (2012a):
			1.56-	8.19	See Schmuck et al. (2001) (DAR)
				3.3	See Stork (1999) (Germany 2005, DAR)
		1–10	4.4-	7.6	Scott-Dupree and Spivak (2001)
	49.4	1–10	1.2		Chauzat et al. (2006)
		1–10	3.3-	3.9	Schmuck et al. (2001)
	0.8	1–10	1.35	<12	Lambert et al. (2013)
		0.1–1		<0.5	Thompson et al. (2013)
Thiacloprid	17.7	100–1,000	75.1	1,002.2	Sanchez-Bayo and Goka (2014)
	62	1–1,000	89.1	1,002.2	Pohorecka et al. (2012)
	2	1–1,000	187.6	326	Rennich et al. (2012)
	5.4	1–1,000	23.8	115	Mullin et al. (2010)
	1.3	1–100	22.3	68	Stoner and Eitzer (2013)
		1–1,000			In EFSA (2012a):
			150-	277	See Von der Ohe (DAR)
			9-	36	See Schatz and Wallner (2009) (DAR)
		1–100	10 to 30	90	Skerl et al. (2009)
Thiamethoxam	12.8	10–1,000	28.9	127	Sanchez-Bayo and Goka (2014)
		0.1–1,000	0.1 to 95.2 + 26.8^h^	127 + 35.1^h^	Dively and Kamel (2012)
	0.3 %	10–100	53.3	53.3	Mullin et al. (2010)
		1–100	12	35	Stoner and Eitzer (2012)
	37	1–10	3.8	9.9	Pohorecka et al. (2012)
	1	1–10	2.8	4.1	Stoner and Eitzer (2013)
		1–100	3^i^ to 7^i^	12	Pilling et al. (2014)
		1–100	1.7	6.2 to 20.4	Krupke et al. (2012)
		1–100			In EFSA (2013b):
			13.41-	21.51	See estimate for maize
			2.37-	3.02	See estimate for sunflower
			4.59-	19.29	See estimate for rape
			4-	12	See Hecht-Rost (2007); Hargreaves (2007) (DAR)
		1–10	2.3 to 2.7		Thompson et al. (2013)
		0.1–10			In EFSA (2012a):
			2.5-	4.2	See Schuld (2001a) (DAR)
				4.6	See Schuld (2001b) (DAR)
				3.6	See Barth (2001) (DAR)
				1.1	See Balluf (2001) (DAR)
				3.2	See Schur (2001c) (DAR)
6-CNA	33	0.1–10	1.2	9.3	Chauzat et al. (2011)
	57.3	0.1–10	1.2		Chauzat et al. (2009)
	44.4	0.1–10	1.2		Chauzat et al. (2006)
Fipronil	2.8 and 3.7^m^	1–100	1.6	29	Sanchez-Bayo and Goka (2014)
	0.3	1–100	28.5	28.5	Mullin et al. (2010)
	6.5	0.1–10	1.2 + 1.0 + 1.7^m^	0.3 + 1.5 + 3.7^m^	Chauzat et al. (2011)
	0.6	1–10	2.8	3.5	Stoner and Eitzer (2013)
	3.7^m^	1–10	2 to 2.3^m^	4	Bernal et al. (2010)
	49^m^	0.1–10	0.8^m^	8.3^m^	Bonmatin et al. (2007)
	12.4	0.1–10	1.2	1.2 + 1.7 + 1^m^	Chauzat et al. (2009)
		1–10		1.9 and 6.4	In EFSA (2013d): see Kerl (2005) (DAR)

6-CNA (6-chloro-nicotinic acid)

^a^Active ingredient

^b^Proportion of positive analyses (see text)

^c^Classified by decade

^d^Mean value from positive analyses

^e^The lowest value of quantified data is followed by a hyphen, the highest value is in the next column

^f^The highest value of quantified data

^g^The sources are related to the original works for avoiding data duplications, and data from DARs (draft assessment report) are available in the cited EFSA reviews

^h^Clothianidin issuing from thiamethoxam

^i^Median values

^j^When data include the UF (1-methyl-3-(tetrahydro-3-furylmethyl)urea) derivative

^k^When data include the derivatives of imidacloprid (olefin, 5-OH, urea, desnitro olefin, desnitro HCl, and 6-CNA)

^l^When data include the derivatives of imidacloprid (5-OH, olefin, or 6-CNA)

^m^Data include some fipronil derivatives (sulfone-, sufide-, or desulfynyl-fipronil)

**Table 5:** Residues (neonicotinoids and fipronil) in nectar or in nectar-derived matrices (nectar/honey)

Insecticide^a^	Detection rate^b^ (%)	Range^c^ (ng/g)	Mean^d^ or magnitude^e,f^ (ng/g)	Maximum^f^ (ng/g)	Reference^g^
Acetamiprid	51	0.1–100	2.4	13.3	Sanchez-Bayo and Goka (2014); Pohorecka et al. (2012)
		0.1–1,000		112.8	Paradis et al. (2014)
Clothianidin	17	0.1–10	1.9	10.1	Sanchez-Bayo and Goka (2014)
		0.1–100	0.1^h^ to 4^h^	12.2^h^	Dively and Kamel (2012)
	17	1–10	2.3	10.1	Pohorecka et al. (2012)
		0.1–10	0.9-	2.2	Cutler and Scott-Dupree (2007); Johnson et al. (2010)
		0.1–1	1^i^	1	Pilling et al. (2014)
	100	10–1,000	89-	319	Larson et al. (2013)
		0.1–100	5	16	Thompson et al. (2013)
		0.1–10	1-	3	Wallner (2009)
		0.1–10			In EFSA (2012a):
			1.2-	8.6	See Schmuk and Shöening (2000a) (DAR)
			0.3-	1	See Maus and Schöening (2002a) (DAR)
			2.8-	3	See Maus and Schöening (2001b) (DAR)
				5.4	See Maus and Schöening (2001c) (DAR)
		0.1–10	0.9-	3.7	Scott-Dupree and Spivak (2001)
		0.1–10	0.32		EFSA (2013a) (estimate)
Dinotefuran		1–100	13.7	21.6	Sanchez-Bayo and Goka (2014)
	100	1–100	2.1 + 0.1^j^ to 9.2 + 4.1^j^	10.8 + 10.8^j^	Dively and kamel (2012)
Imidacloprid	21.4	1–100	6	72.8	Sanchez-Bayo and Goka (2014)
		10–100	13.37 to 72.81	95.2	Byrne et al. (2014)
		0.1–100	0.1 to 11.2 + 6.4^k^	13.7 + 9.4^k^	Dively and Kamel (2012)
	21.8	0.1–10	0.7	1.8	Chauzat et al. (2011)
		100–1,000		660^j^	Paine et al. (2011)
		100–1,000		171	Larson et al. (2013)
		1–100	6.6 + 1.1 + 0.2^l^	16 + 2.4 + 0.5^l^	Krischik et al. (2007)
		0.1–100	0.1 to 11.2 + 6.4^k^	13.7 + 9.4^k^	Dively and Kamel (2012)
		1–100	10.3	14	Stoner and Eitzer (2012)
		1–10			In EFSA (2012a):
			3.45-	4.6	See Stork (1999) (DAR)
			1.59-	8.35	See Germany (2005) (DAR)
	29.7	0.1–10	0.7 + 1.2^l^		Chauzat et al. (2009)
		0.1–10	1.9		Schmuck et al. (2001)
	21	0.1–10	0.6	2	Pohorecka et al. (2012)
		0.1–10	0.2^l^-	3.9^l^	Wiest et al. (2011)
	2.1	0.1–10	0.14^l^	<3.9^l^	Lambert et al. (2013)
		0.1–1	0.6-	0.8	Scott-Dupree and Spivak (2001)
		0.1–1	0.45	0.5	Thompson et al. (2013)
Thiacloprid	64	1–1,000	6.5	208.8	Sanchez-Bayo and Goka (2014); Pohorecka et al. (2012)
		1–100	1.8	36	Schatz and Wallner (2009)
		1–100		33	Johnson et al. (2010)
		1–100		11.6	Paradis et al. (2014)
Thiamethoxam	65	0.1–100	6.4	17	Sanchez-Bayo and Goka (2014)
		0.1–100	0.1 to 9.5 + 4^h^	12.2 + 6.4^h^	Dively and Kamel (2012)
	65	0.1–100	4.2	12.9	Pohorecka et al. (2012)
		0.1–10	0.7 to 2.4^i^ + 1^i^	4,7 + 1	Pilling et al. (2014)
		1–100	11	20	Stoner and Eitzer (2012)
		0.1–10	0.59	4	EFSA (2013b): see Hecht-Rost (2007) (DAR)
		0.1–10		1.5 and 3.9	Thompson et al. (2013)
		0.1–10	0.65	2.72	EFSA (2013e) (estimate)
		0.1–10		2	Paradis et al. (2014)
		0.1–10			In EFSA (2012a):
			1.0	2.1	See Shuld (2001a) (DAR)
				0.9	See Purdy (2000) (DAR)
				1	See Balluf (2001) (DAR)
6-CNA	17.6	0.1–10	1.2	10.2	Chauzat et al. (2011)
Fipronil	6.5	10–100	70	100	Pareja et al. (2011)
	0.3	10–100	28.5		Mullin et al. (2010)
		0.1–10			In EFSA (2013d):
			2.3	6.4	See Kerl (2005) (DAR)
				3.3	See Bocksch (2009) (DAR)

6-CNA (6-chloro-nicotinic acid)

^a^Active ingredient

^b^Proportion of positive analyses (see text)

^c^Classified by decade

^d^Mean value from positive analyses

^e^The lowest value of quantified data is followed by a hyphen, the highest value is in the next column

^f^The highest value of quantified data

^g^The sources are related to the original works for avoiding data duplications, and data from DARs (draft assessment report) are available in the cited EFSA reviews

^h^Clothianidin issuing from thiamethoxam

^i^Median values

^j^When data include the UF (1-methyl-3-(tetrahydro-3-furylmethyl)urea) derivative

^k^When data include the derivatives of imidacloprid (olefin, 5-OH, urea, desnitro olefin, desnitro HCl, and 6-CNA)

^l^When data include the derivatives of imidacloprid (5-OH, olefin, or 6-CNA)

According to a global analysis by Sanchez-Bayo and Goka (2014), which does not distinguish between the routes of exposure, crop species, or the mode of insecticide application, the detection rate of various agrochemicals in pollen/beebread were as follows: acetamiprid at 24 %, thiacloprid at 18 %, imidacloprid at 16 %, thiamethoxam at 13 %, clothianidin at 11 %, fipronil at 3 %, and dinotefuran at 1 % (although Dively and Kamel (2012) reported 100 % for dinotefuran). While the active ingredients were not detected or quantified in most of the samples analyzed, the results also show that the oldest measurements often had the lowest occurrence rate, confirming the influence of the sensitivity of analytical techniques on this parameter.

Interestingly, the maximum residue levels in Table 4 are thiacloprid (1,002 ng/g), imidacloprid (912 ng/g), dinotefuran (168 ng/g), acetamiprid (134 ng/g), thiamethoxam (127 ng/g), clothianidin (41 ng/g), and fipronil (29 ng/g). For each of these compounds, these values must be interpreted with respect to the corresponding data for toxicity. However, these values represent the worst case scenarios. Further examination of exposure data shows that average levels in pollen/beebread are lower than these maximums, due to some data issuing from various types of application techniques (soil treatment, injection, spray, seed dressing, etc.). For example, it has been reported that aerial treatments represent a significantly higher source of contamination than seed-dressing treatments (Thompson 2012; EFSA 2012a). This explains the high variability of results when concentrations are ranked by decades. However, when imidacloprid was used as a seed dressing, mean residue levels were mostly found to be in the range of 1–10 ng/g and variability among crops was not so high (sunflower, maize, and canola), whereas spray or soil application led to higher values, by 1 order of magnitude. To a lesser extent, this was also observed for clothianidin and thiamethoxam. Therefore, averaged data must also be considered to gain a better idea of the average contamination of pollen/beebread: thiacloprid (75 ng/g), dinotefuran (45 ng/g), thiamethoxam (29 ng/g), imidacloprid (20 ng/g), clothianidin (9 ng/g), acetamiprid (3 ng/g), and fipronil (1.6 ng/g) (Sanchez-Bayo and Goka 2014). As a consequence, the latter values are the most relevant for toxicity studies for nontarget species.

##### Nectar and honey

The work conducted by the EFSA (2012b) generally reported lower neonicotinoid concentrations in nectar than in pollen (see also Goulson 2013). Data reported by scientific reviews, scientific literature, and some relevant DARs are presented in Table 5 (Thompson 2012; EFSA 2012a, 2013a, b, d, e; Sanchez-Bayo and Goka 2014). Relatively recent reviews were done for the purpose of assessing neonicotinoids and fipronil. According to a global analysis by Sanchez-Bayo and Goka (2014), thiamethoxam was detected in 65 % of nectar/honey samples, followed by thiacloprid at 64 %, acetamiprid at 51 %, imidacloprid at 21 %, clothianidin at 17 %, and fipronil at 6.5 %. Note that the study of Dively and Kamel (2012) showed that dinotefuran was always detected (100 %) in pumpkin nectar samples in 2009. Contrary to the pollen/beebread case, three neonicotinoids were found in most of the nectar/honey from treated crops (Sanchez-Bayo and Goka 2014). However, the higher proportion of neonicotinoids in nectar/honey than in pollen/beebread could be linked to the higher sensitivity of the analytical techniques used. Validation of analytical methods for nectar/honey generally lead to LOD and LOQ values which are lower than in the case of pollen/beebread (Mullin et al. 2010; Lambert et al. 2013; Thompson et al. 2013), the latter being a difficult matrix to analyze due to the encapsulated nature of pollen and other interferences.

The values of Sanchez-Bayo and Goka (2014) for maximum levels in nectar/honey are thiacloprid (209 ng/g), imidacloprid (73 ng/g), dinotefuran (22 ng/g), thiamethoxam (17 ng/g), acetamiprid (13 ng/g), and clothianidin (10 ng/g). From these data, it appears that nectar/honey is significantly less contaminated than pollen/beebread, by a factor of 4 (clothianidin) to 12 (imidacloprid). Note that very recently, Paradis et al. (2014) reported a maximum of 112.8 ng/g in nectar for acetamiprid, Larson et al. (2013) reported 319 ng/g for clothianidin, Paine et al. (2011) reported 660 ng/g for imidacloprid, and Pareja et al. (2011) measured 100 ng/g for fipronil. The maximum level of fipronil in nectar/honey is three times higher than that in pollen/beebread, despite the fact that fipronil is less water soluble than the neonicotinoids. Obviously, these levels must be interpreted with respect to the corresponding toxicity data for each of these compounds. Another study by Kasiotis et al. (2014) measured a maximum residue level of imidacloprid of 73.9 ng/g, this value being similar to the 95.2 ng/g value detected by Byrne et al. (2014). The maximum for imidacloprid was found to be 41,273 ng/g by Kasiotis et al. (2014); however, it should be noted that some sampling was conducted directly by beekeepers after bee collapse incidents, so it is possible that external contamination may have occurred (data not included in Table 5). As with the residue levels in pollen and bee bread, these values represent a worst case situation and do not give a general measure of contamination.

Table 5 shows that average residue levels in nectar/honey are significantly lower than the above maximums, again due to the data issuing from various types of application techniques (soil drench, injection, spray, seed dressing, etc.). Again, aerial treatments represent a significantly higher source of contamination in nectar/honey than when used as a seed dressing (Thompson 2012; EFSA 2012a). This explains the high variability of results when concentrations are ranked by decades, as observed for imidacloprid for instance. Similar to the case of pollen/beebread, imidacloprid used as seed dressing led to levels mainly in the range of 1–10 ng/g (sunflower, cotton, and canola; EFSA 2013c), but soil application on eucalyptus led to higher values by 2 orders of magnitude (Paine et al. 2011). That is why averaged data are also to be considered: dinotefuran (13.7 ng/g), thiacloprid (6.5 ng/g), thiamethoxam (6.4 ng/g), imidacloprid (6 ng/g), acetamiprid (2.4 ng/g), and clothianidin (1.9 ng/g). As with the maximum levels, it appears that nectar/honey is less contaminated than pollen/beebread by a factor of 1.2 (acetamiprid) to 11.5 (thiacloprid). This further confirms that the first matrix is less contaminated by neonicotinoids than the second one. In the particular case of the study by Kasiotis et al. (2014), mean levels were found to be 48.7 ng/g for imidacloprid and 3,285 ng/g for clothianidin. It is difficult to investigate the particular case of fipronil because data are still lacking and published data are rather heterogeneous. Higher levels of fipronil were measured in nectar/honey than in pollen/beebread.

##### Conclusions

Pollen/beebread and nectar/honey appear to be very relevant routes of exposure to neonicotinoids and fipronil in terms of occurrence, average level, and maximum residue level. The few studies of fipronil provide very heterogeneous results. Pollen/beebread revealed average residue levels between 0.8 and 28.5 ng/g. Nectar/honey revealed average residue levels between 2.3 and 70 ng/g. For neonicotinoids, average residue levels from Sanchez-Bayo and Goka (2014) are in the range of 1.9–13.7 ng/g for nectar/honey, and in the range of 3–75.1 ng/g for pollen/beebread. However, higher values of average residue levels have been obtained in several studies (Tables 4 and 5). Maximum levels of these systemic insecticides were found in the range of 10.1–208.8 ng/g for nectar/honey, and in the range of 29–1,002 ng/g for pollen/beebread (Sanchez-Bayo and Goka 2014). In terms of maximum levels, the variability clearly shows that contamination of pollen and nectar is not predictable and controlled, and that very high residue levels can be found in both pollen and nectar. It is important to note that nontarget species are exposed to more than just one pesticide via pollen or nectar. This was recently exemplified by the detection of mixtures of three to four insecticides (from a pool of 22 insecticides analyzed) in the nectar collected by honey bees, including acetamiprid, thiacloprid, thiamethoxam, tau-fluvalinate, and deltamethin (Paradis et al. 2014). Note that for the latter study, the agricultural uses of fipronil in France had been suspended several years prior, as well as the uses of imidacloprid for sunflower and maize.

Finally, nontarget species are very likely to be exposed to multiple pesticides (insecticides, herbicides, and several fungicides) simultaneously or at different points in time, and via multiple routes including pollen and nectar. This is especially relevant for treated fruit trees. In the cases of neonicotinoids and fipronil, variability of exposure data remains high between and within studies, due to variability of (1) pesticide applications, (2) the crops considered, (3) the samples analyzed, and (4) measurement methods. Variability will be difficult to improve and assess because field trials demand robust protocols that are difficult to manage, and also the required sensitive analytical techniques are costly to utilize. Therefore, despite the large methodological progress that has been made in the last decade, the question of exposure inherently leads to heterogeneous results and remains the object of discussion. Despite this variability, which does not imply inaccuracy of measurements in real situations, studies worldwide demonstrate the exposure of nontarget species to these pesticides. This exposure, specifically through nectar and pollen, has proved harmful for bees and other pollinators (Pisa et al. 2014, this issue).

#### Honeydew

Honeydew is produced mainly by aphids (Aphididae) and other heteropteran insects and consists of a sticky, sugary liquid. Among others, insects such as ants (Formicidae) feed directly on honeydew while insects such as honeybees (*A. mellifera*) and wasps collect honeydew. It may be argued that honeydew production on treated crops is negligible, as the aphids that produce it would not be present on such crops. Van der Sluijs et al. (2013) argue that given the longer life span of bees, concentrations in plant sap that are too low to kill aphids could eventually prove harmful to bees through repeated exposure. However, there is no data available to verify this hypothesis. EFSA (2013d) therefore concludes that honeydew should be taken into account as a potential exposure route for honeybees in the case of fipronil.

## Conclusion

The chemical properties of neonicotinoids and fipronil mean that they have the potential to accumulate in the environment at field-realistic levels of use (Bonmatin et al. 2007). This combination of persistence (over months or years) and solubility in water leads to contamination of, and the potential for accumulation in, soils and sediments (ppb-ppm range), waterways (groundwater and surface water in the ppt-ppb range), and treated and nontreated vegetation (ppb-ppm range) (Goulson 2013).

Screening of these matrices for pesticides is very patchy, and even where it has been conducted, the toxic metabolites are often not included. However, where environmental samples have been screened they are commonly found to contain mixtures of neonicotinoids or fipronil, along with their toxic metabolites and other pesticides. In addition, measurements taken from water have been found to exceed ecotoxicological limits on a regular basis around the globe (e.g., Gonzalez-Pradas et al. 2002; Kreuger et al. 2010; Starner and Goh 2012; Masiá et al. 2013; Van Dijk et al. 2013).

The presence of these compounds in the environment suggests that all kinds of nontarget organisms will be exposed to them. The case of honeybees is very illustrative, as they are exposed from the sowing period until flowering. In spring, the use of seed-coating insecticides for crops poses a risk of acute intoxication for bees (and other pollinators) by direct exposure of flying bees to dusts emitted by the drilling machine (Girolami et al. 2013). The use of spray also exposes nontarget organisms when foraging on flowers, especially on fruit trees. Regardless of the mode of application, bees bring contaminated pollen, nectar, and probably also contaminated water back to the hive. Analysis of residues in food stores of honeybee colonies from across the globe reveal exactly what we might predict, based on the physical and chemical properties of these compounds. These food stores routinely contain mixtures of neonicotinoids and fipronil, generally in the 1–100 ppb range, demonstrating chronic exposure of honeybees throughout their lives (Sanchez-Bayo and Goka 2014). Similar exposure can be expected for other less-studied pollinators and invertebrates. Such widespread contamination has an impact on both aquatic and terrestrial invertebrates (Pisa et al. 2014, this issue) and vertebrates (Gibbons et al. 2014, this issue) living in or near farmland, or in streams which may occur in proximity to farmed areas.

This environmental contamination will undoubtedly have impacts on the functioning of various ecosystems and their services (Chagnon et al. 2014, this issue) unless alternatives are developed (Furlan and Kreutzweiser 2014, this issue; Van der Sluijs et al. 2014, this issue).
